# Supplementary material for: Arthropod biodiversity loss from nitrogen deposition is buffered by natural and semi-natural habitats
Source: PLoS Biol. 2025 Jul 22;23(7):e3003285. doi: 10.1371/journal.pbio.3003285 (PMC12282910; doi:10.1371/journal.pbio.3003285)
Supplement: S1 Fig — N deposition values were loge-transformed, with oxidized N deposition ranging from 0.7 mg N m−2 yr−1 to 3220.6 mg N m−2 yr−1, reduced nitrogen deposition ranging from 0.0 mg N m−2 yr−1 to 6576.8 mg N m−2 yr−1. The outline map is based on the Natural Earth (http://www.naturalearthdata.com/about/terms-of-use/), which is published under a CC-BY 4.0 license. The data underlying this figure can be found in link: https://thredds.met.no/thredds/catalog/data/EMEP/Articles_data/Schwede_etal_Ndep_2018/catalog.html. (DOCX) [file pbio.3003285.s001.docx]

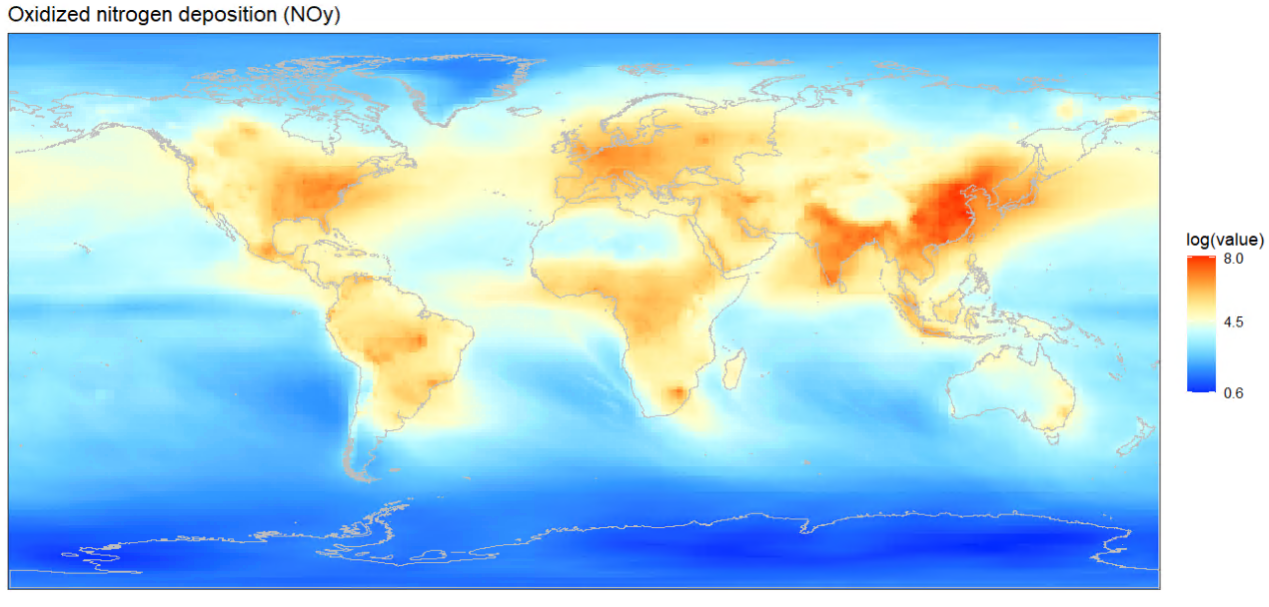


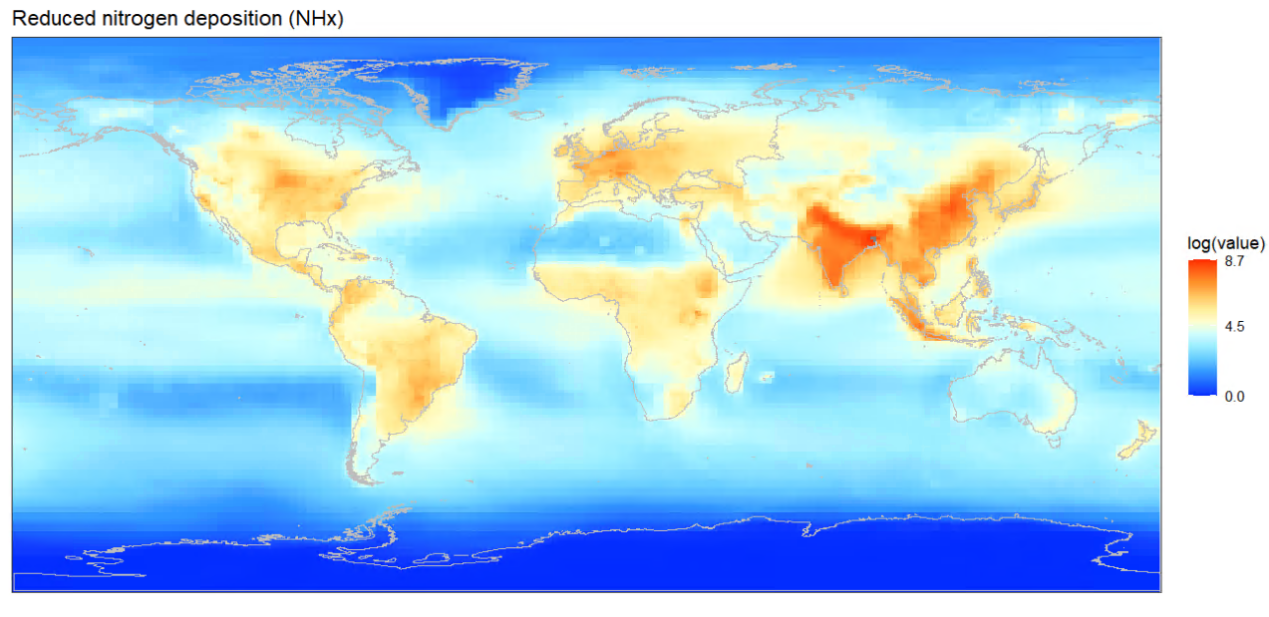


**S1 Fig. Distribution of** **oxidized nitrogen deposition (NOy) and** **reduced nitrogen deposition (NHx) globally.** N deposition values were log_e_-transformed, with oxidized N deposition ranging from 0.7 mg N m^-2^ yr^-1^ to 3220.6 mg N m^-2^ yr^-1^, reduced nitrogen deposition ranging from 0.0 mg N m^-2^ yr^-1^ to 6576.8 mg N m^-2^ yr^-1^. The outline map is based on Natural Earth Data (http://www.naturalearthdata.com/about/terms-of-use/), which is published under a CC-BY 4.0 license. The data underlying this Figure can be found in link: https://thredds.met.no/thredds/catalog/data/EMEP/Articles_data/Schwede_etal_Ndep_2018/catalog.html.
