# Supplementary material for: Arthropod biodiversity loss from nitrogen deposition is buffered by natural and semi-natural habitats
Source: PLoS Biol. 2025 Jul 22;23(7):e3003285. doi: 10.1371/journal.pbio.3003285 (PMC12282910; doi:10.1371/journal.pbio.3003285)
Supplement: S2 Fig — The outline map is based on the Natural Earth (http://www.naturalearthdata.com/about/terms-of-use/), which is published under a CC-BY 4.0 license. The data underlying this figure can be found in https://doi.org/10.6084/m9.figshare.29109170. (DOCX) [file pbio.3003285.s002.docx]

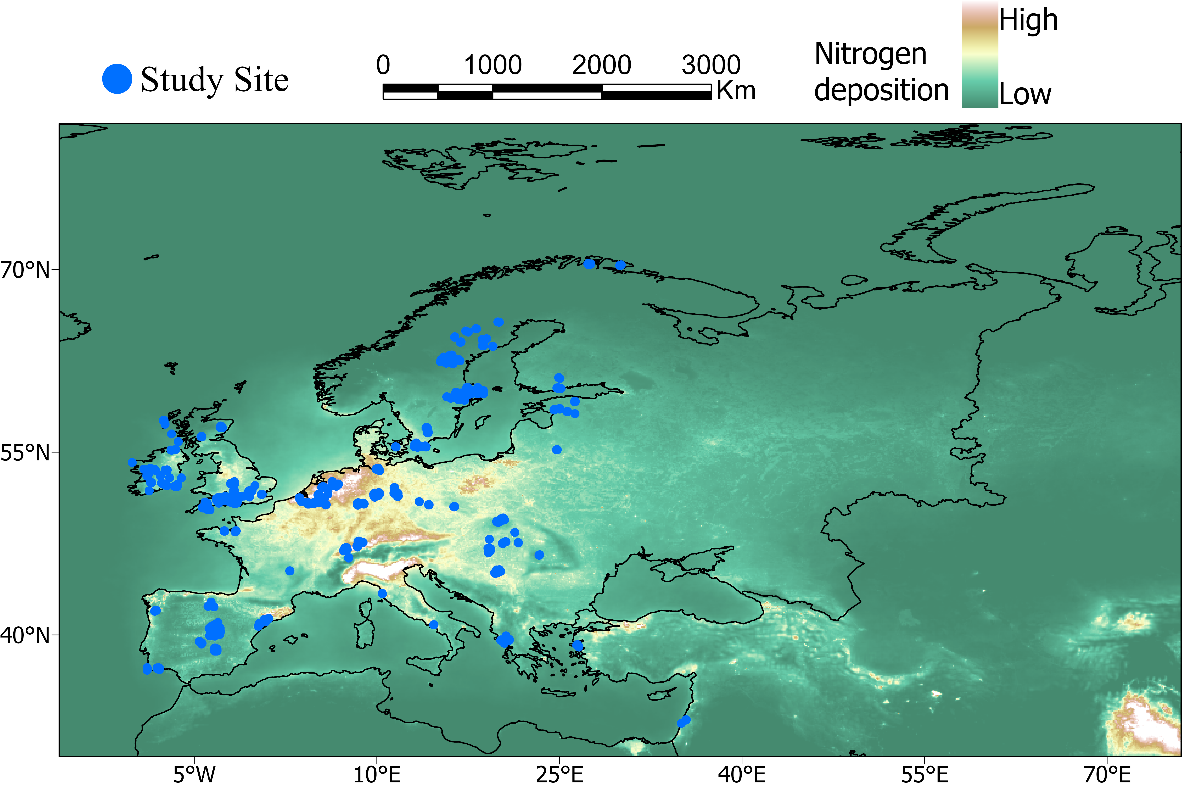


**S2 Fig. Distribution of N deposition sampling sites in Europe**. The outline map is based on the Natural Earth data (http://www.naturalearthdata.com/about/terms-of-use/), which is published under a CC-BY 4.0 license. The data underlying this Figure can be found in DOI:10.6084/m9.figshare.29109170.
