# Supplementary material for: Arthropod biodiversity loss from nitrogen deposition is buffered by natural and semi-natural habitats
Source: PLoS Biol. 2025 Jul 22;23(7):e3003285. doi: 10.1371/journal.pbio.3003285 (PMC12282910; doi:10.1371/journal.pbio.3003285)
Supplement: S3 Fig — Values represent the percentage difference compared with Primary vegetation with the 2.5th percentile value of total N deposition among sampled sites. The lines with different colors represent the median predicted value for each land-use type, with shaded areas representing the 95% confidence intervals. The results are predicted across 95% of the range of N deposition values for each land-use type. The number of sites were: Primary vegetation, Nsite = 1,094; Secondary vegetation, Nsite = 1,136; Cropland, Nsite = 1,285; Pasture, Nsite = 989; Plantation forest, Nsite = 277. The N deposition range used for plotting covers from 2.5% to 97.5% of sampled sites for each of the land-use types included in the model: 109.4–2326.3 mg N m−2 yr−1. The data underlying this figure can be found in https://doi.org/10.6084/m9.figshare.29109170. (DOCX) [file pbio.3003285.s003.docx]

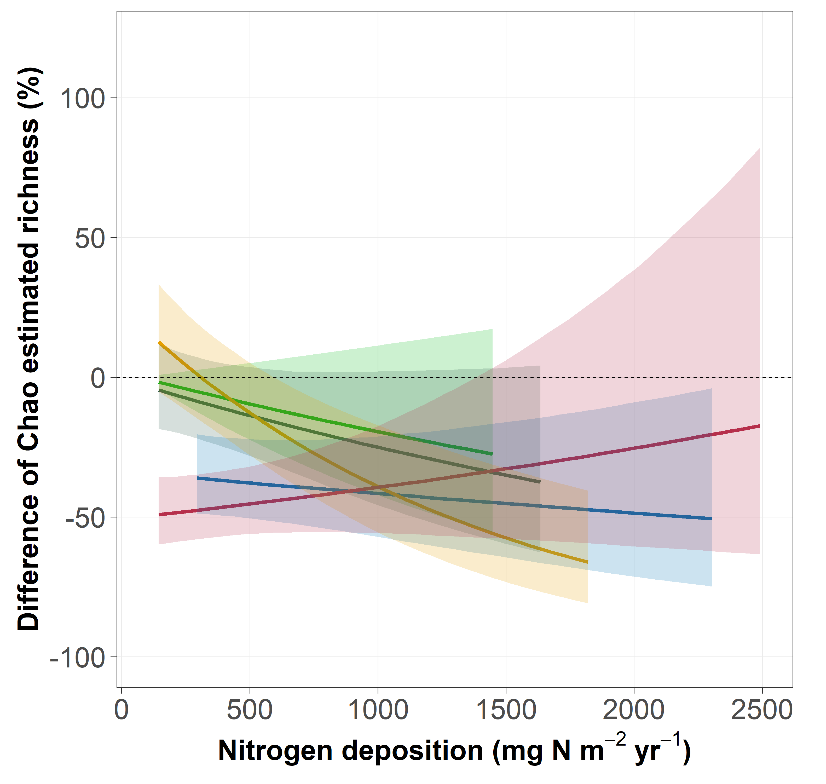


**S3 Fig. The effects of the total N deposition interact with land use on Arthropods Chao estimated species richness**. Values represent the percentage difference compared with primary vegetation with the 2.5th percentile value of total N deposition among sampled sites. The lines with different colors represent the median predicted value for each land-use type, with shaded areas representing the 95% confidence intervals. The results are predicted across 95% of the range of N deposition values for each land-use type. The number of sites were: Primary vegetation, N_site_ = 1,094; Secondary vegetation, N_site_ = 1,136; Cropland, N_site_ = 1,285; Pasture, N_site_ = 989; Plantation forest, N_site_ = 277. The N deposition range used for plotting covers from 2.5% to 97.5% of sampled sites for each of the land-use types included in the model: 109.4 to 2326.3 mg N m^-2^ yr^-1^. The data underlying this Figure can be found in DOI:10.6084/m9.figshare.29109170.
