# Supplementary material for: Arthropod biodiversity loss from nitrogen deposition is buffered by natural and semi-natural habitats
Source: PLoS Biol. 2025 Jul 22;23(7):e3003285. doi: 10.1371/journal.pbio.3003285 (PMC12282910; doi:10.1371/journal.pbio.3003285)
Supplement: S5 Fig — A, fitted versus residuals plot to check for constant variance across the range of fitted values. B is Normal QQ-plot to check for a normal distribution of residuals. C is distribution of P-values from sets of Moran’s I test for spatial autocorrelation in the residuals for each study. Red line represents a P-value of 0.05. The left of red line represents the studies with significant spatial autocorrelation. D is observed values versus fitted values. The data underlying this figure can be found in https://doi.org/10.6084/m9.figshare.29109170. (DOCX) [file pbio.3003285.s005.docx]

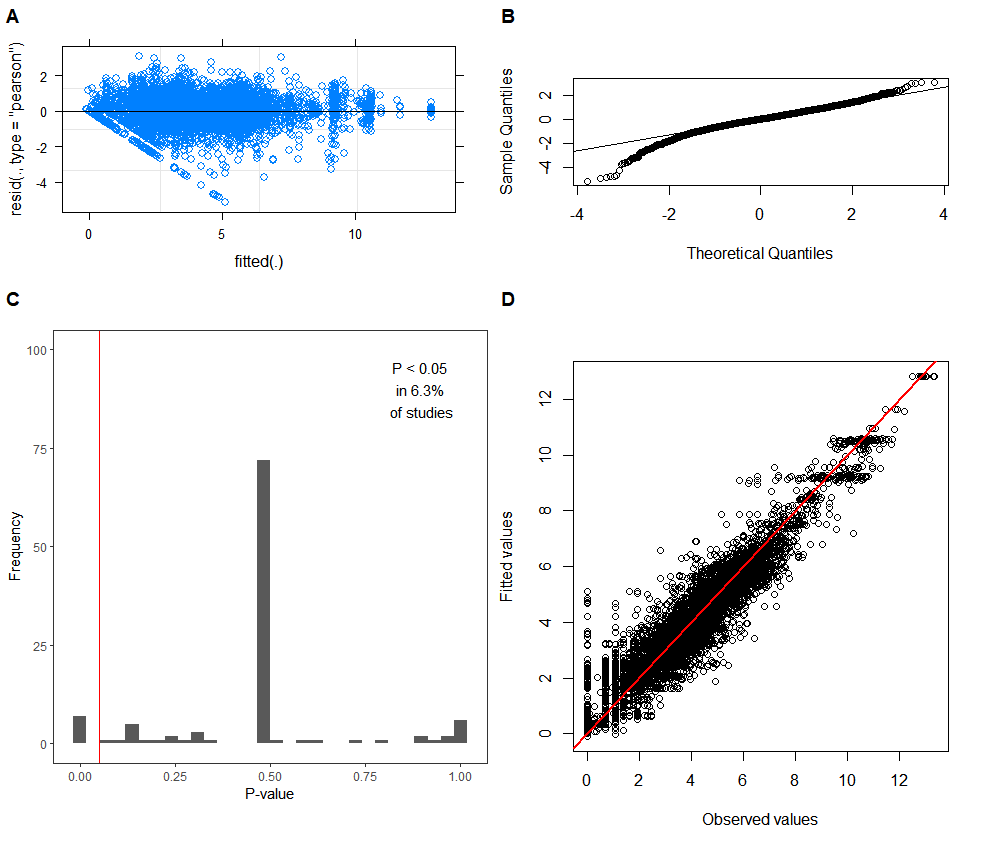


**S5 Fig.** **Model checks for the final total abundance model obtained through backward stepwise selection for the study.** A, fitted versus residuals plot to check for constant variance across the range of fitted values. B is Normal QQ-plot to check for a normal distribution of residuals. C is distribution of P values from sets of Moran’s I test for spatial autocorrelation in the residuals for each study. Red line represents a P value of 0.05. The left of red line represents the studies with significant spatial autocorrelation. D is observed values versus fitted values. The data underlying this Figure can be found in DOI:10.6084/m9.figshare.29109170.
