# Supplementary material for: Arthropod biodiversity loss from nitrogen deposition is buffered by natural and semi-natural habitats
Source: PLoS Biol. 2025 Jul 22;23(7):e3003285. doi: 10.1371/journal.pbio.3003285 (PMC12282910; doi:10.1371/journal.pbio.3003285)
Supplement: S1 Table — (DOCX) [file pbio.3003285.s006.docx]

**S1 Table: likelihood test result for species richness model**

| **Item** | **Chisq** | **Df** | **Pr(>Chisq)** |
| --- | --- | --- | --- |
| (Intercept) | 810.6762 | 1 | **< 2.2e-16** |
| Predominant_land_use | 218.2440 | 4 | **< 2.2e-16** |
| pnhRS | 10.7857 | 1 | **0.0010229** |
| crpRS | 8.4590 | 1 | **0.0036325** |
| NDRS | 18.8630 | 1 | **1.404e-05** |
| tmpRS | 2.6766 | 1 | 0.1018308 |
| NDRS:tmpRS | 8.4707 | 1 | **0.0036092** |
| crpRS:NDRS | 15.0823 | 1 | **0.0001029** |
| Predominant_land_use:NDRS | 35.6271 | 4 | **3.453e-07** |
| Predominant_land_use:pnhRS:NDRS | 40.0908 | 5 | **1.432e-07** |

**Species_richness ~ Predominant_land_use+pnhRS+ NDRS:tmpRS+ NDRS:crpRS+ NDRS:Predominant_land_use+NDRS:Predominant_land_use:pnhRS+NDRS+tmpRS+crpRS + (1|SS) + (1|SSB) + (1|SSBS)**
