# Supplementary material for: Arthropod biodiversity loss from nitrogen deposition is buffered by natural and semi-natural habitats
Source: PLoS Biol. 2025 Jul 22;23(7):e3003285. doi: 10.1371/journal.pbio.3003285 (PMC12282910; doi:10.1371/journal.pbio.3003285)
Supplement: S2 Table — (DOCX) [file pbio.3003285.s007.docx]

**S2 Table: likelihood test result for total abundance model**

| **Item** | **Chisq** | **Df** | **Pr(>Chisq)** |
| --- | --- | --- | --- |
| (Intercept) | 1062.1890 | 1 | **< 2.2e-16** |
| Predominant_land_use | 98.9162 | 4 | **< 2.2e-16** |
| crpRS | 13.7971 | 1 | **0.0002036** |
| NDRS | 17.4704 | 1 | **2.918e-05** |
| tmpRS | 7.3705 | 1 | 0.0066303 |
| pnhRS | 0.1920 | 1 | 0.6612603 |
| NDRS:crpRS | 9.4916 | 1 | **0.0020642** |
| Predominant_land_use:NDRS | 30.6824 | 4 | **3.554e-06** |
| Predominant_land_use:NDRS:pnhRS | 33.8165 | 5 | **2.590e-06** |

**logAbun ~ Predominant_land_use+pnhRS+ NDRS:crpRS+NDRS:Predominant_land_use+NDRS:Predominant_land_use:pnhRS+NDRS+tmpRS+crpRS + (1|SS) + (1|SSB)**
