# Supplementary material for: Arthropod biodiversity loss from nitrogen deposition is buffered by natural and semi-natural habitats
Source: PLoS Biol. 2025 Jul 22;23(7):e3003285. doi: 10.1371/journal.pbio.3003285 (PMC12282910; doi:10.1371/journal.pbio.3003285)
Supplement: S3 Table — Output mainly includes two parts: fixed part and random part. Fixed part includes estimates, 95% confidence intervals (CI) and p-values. Random part include: τ00 is the model variance explained by the random effects (SS represent studies, SSB represent blocks, SSBS represent sites within blocks), σ2 is the residual variance, and the marginal and conditional R2 values. (DOCX) [file pbio.3003285.s008.docx]

|  | **Species_richness** | | |
| --- | --- | --- | --- |
| Predictors | Estimates | CI | P |
| **Fixed Effects** |  |  |  |
| (Intercept) | 2.45 | 2.28 – 2.62 | **<0.001** |
| Predominant land use [Primary vegetation] | 0.33 | 0.27 – 0.40 | **<0.001** |
| Predominant land use [Secondary vegetation] | 0.22 | 0.16 – 0.29 | **<0.001** |
| Predominant land use [Pasture] | 0.12 | 0.05 – 0.19 | **0.001** |
| Predominant land use [Plantation forest] | -0.13 | -0.22 – -0.03 | **0.007** |
| pnhRS | 0.07 | 0.03 – 0.11 | **0.001** |
| crpRS | -0.05 | -0.08 – -0.01 | **0.004** |
| NDRS | -0.26 | -0.37 – -0.14 | **<0.001** |
| tmpRS | -0.09 | -0.21 – 0.02 | 0.102 |
| NDRS × tmpRS | -0.15 | -0.25 – -0.05 | **0.004** |
| crpRS × NDRS | 0.06 | 0.03 – 0.10 | **<0.001** |
| Predominant land use [Primary vegetation] ×NDRS | 0.12 | 0.03 – 0.20 | **0.009** |
| Predominant land use [Secondary vegetation] ×NDRS | 0.16 | 0.07 – 0.24 | **<0.001** |
| Predominant land use [Pasture] × NDRS | 0.09 | 0.02 – 0.15 | **0.011** |
| Predominant land use [Plantation forest] ×NDRS | 0.31 | 0.20 – 0.42 | **<0.001** |
| (Predominant land use[Cropland] × pnhRS) ×  NDRS | -0.03 | -0.10 – 0.04 | 0.409 |
| (Predominant land use [Primary vegetation] × pnhRS) × NDRS | 0.16 | 0.10 – 0.22 | **<0.001** |
| (Predominant land use [Secondary vegetation] × pnhRS) × NDRS | 0.11 | 0.05 – 0.17 | **0.001** |
| (Predominant land use [Pasture] × pnhRS) × NDRS | 0.03 | -0.04 – 0.11 | 0.392 |
| (Predominant land use [Plantation forest] × pnhRS) × NDRS | 0.12 | 0.05 – 0.19 | **0.001** |
| **Random Effects** |  |  |  |
| σ^2^ | 0.07 |  |  |
| τ_00 SSBS_ | 0.07 |  |  |
| τ_00 SSB_ | 0.05 |  |  |
| τ_00 SS_ | 1.63 |  |  |
| Marginal R^2^/ Conditional R^2^ | 0.030/0.960 |  |  |

**S3 Table: The effects of the total nitrogen deposition on species richness of Arthropods.** Output mainly includes two parts: fixed part and random part. Fixed part includes estimates, 95% confidence intervals (CI) and *p* values. Random part include: τ_00_ is the model variance explained by the random effects (SS represent studies, SSB represent blocks, SSBS represent sites within blocks), σ^2^ is the residual variance, and the marginal and conditional R^2^ values.
