# Supplementary material for: Arthropod biodiversity loss from nitrogen deposition is buffered by natural and semi-natural habitats
Source: PLoS Biol. 2025 Jul 22;23(7):e3003285. doi: 10.1371/journal.pbio.3003285 (PMC12282910; doi:10.1371/journal.pbio.3003285)
Supplement: S9 Table — Output mainly includes two parts: fixed part and random part. Fixed part includes estimates, 95% confidence intervals (CI) and p-values. Random part includes: τ00 for the model variance explained by the random effects (SS represent studies, SSB represent blocks, SSBS represent sites within blocks), σ2 for the residual variance, and the marginal and conditional R2 values. The oxnrdnRS represent the nitrogen deposition data derived from EMEP model. (DOCX) [file pbio.3003285.s014.docx]

**S9 Table: The effects of nitrogen deposition on the species richness of Arthropods in Europe with a finer resolution dataset (0.1°×0.1°).** Output mainly includes two parts: fixed part and random part. Fixed part includes estimates, 95% confidence intervals (CI) and *p* values. Random part includes: τ_00_ for the model variance explained by the random effects (SS represent studies, SSB represent blocks, SSBS represent sites within blocks), σ^2^ for the residual variance, and the marginal and conditional R^2^ values. The oxnrdnRS represent the nitrogen deposition data derived from EMEP model.

|  | **Species_richness** | | |
| --- | --- | --- | --- |
| Predictors | Estimates | CI | P |
| **Fixed Effects** |  |  |  |
| (Intercept) | 1.84 | 1.35 – 2.32 | **<0.001** |
| Predominant land use [Primary vegetation] | 0.19 | -0.01 – 0.39 | 0.067 |
| Predominant land use [Secondary vegetation] | 0.20 | 0.03 – 0.36 | **0.024** |
| Predominant land use [Pasture] | 0.23 | 0.12 – 0.34 | **<0.001** |
| Predominant land use [Plantation forest] | -0.69 | -1.54 – 0.15 | 0.109 |
| pnhRS | 0.04 | -0.05 – 0.12 | 0.376 |
| crpRS | -0.03 | -0.08 – 0.02 | 0.271 |
| oxnrdnRS | -0.49 | -0.83 – -0.15 | **0.005** |
| tmpRS | -0.69 | -1.17 – -0.21 | **0.005** |
| oxnrdnRS × tmpRS | -0.28 | -0.67 – 0.11 | 0.164 |
| crpRS × oxnrdnRS | 0.04 | -0.02 – 0.11 | 0.183 |
| Predominant land use [Primary vegetation] × oxnrdnRS | 0.35 | 0.10 – 0.60 | **0.006** |
| Predominant land use [Secondary vegetation] × oxnrdnRS | 0.29 | 0.08 – 0.50 | **0.007** |
| Predominant land use [Pasture] × oxnrdnRS | 0.30 | 0.17 – 0.43 | **<0.001** |
| Predominant land use [Plantation forest] × oxnrdnRS | -0.55 | -1.81 – 0.72 | 0.399 |
| (Predominant land use [Cropland] × pnhRS) × oxnrdnRS | -0.03 | -0.19 – 0.13 | 0.705 |
| (Predominant land use [Primary vegetation] × pnhRS) × oxnrdnRS | 0.14 | -0.04 – 0.32 | 0.121 |
| (Predominant land use [Secondary vegetation] × pnhRS) × oxnrdnRS | -0.00 | -0.12 – 0.12 | 0.969 |
| (Predominant land use [Pasture] × pnhRS) × oxnrdnRS | 0.21 | 0.09 – 0.32 | **0.001** |
| (Predominant land use [Plantation forest] × pnhRS) × oxnrdnRS | 0.21 | -0.60 – 1.02 | 0.604 |
| **Random Effects** |  |  |  |
| σ^2^ | 0.06 |  |  |
| τ_00 SSBS_ | 0.06 |  |  |
| τ_00 SSB_ | 0.05 |  |  |
| τ_00 SS_ | 2.06 |  |  |
| Marginal R^2^/ Conditional R^2^ | 0.040/0.973 |  |  |
