# Supplementary material for: Arthropod biodiversity loss from nitrogen deposition is buffered by natural and semi-natural habitats
Source: PLoS Biol. 2025 Jul 22;23(7):e3003285. doi: 10.1371/journal.pbio.3003285 (PMC12282910; doi:10.1371/journal.pbio.3003285)
Supplement: S12 Table — (DOCX) [file pbio.3003285.s017.docx]

**S12 Table: Variance inflation factors for the explanatory variables in richness model**

| **Variable** | **VIF** |
| --- | --- |
| NDRS | 1.499619 |
| pnhRS | 1.298209 |
| crpRS | 1.374028 |
| tmpRS | 1.017790 |
| Predominant_land_use | 2.220641 |
| NDRS:tmpRS | 1.201760 |
| NDRS:crpRS | 1.457207 |
| NDRS:Predominant_land_use | 3.157689 |
| NDRS:Predominant_land_use:pnhRS | 3.848723 |

**Model formula**: Species_richness ~ Predominant_land_use+pnhRS+ NDRS:tmpRS+ NDRS:crpRS+NDRS:Predominant_land_use+NDRS:Predominant_land_use:pnhRS+NDRS+tmpRS+crpRS + (1|SS) + (1|SSB) + (1|SSBS)
