# Supplementary material for: Arthropod biodiversity loss from nitrogen deposition is buffered by natural and semi-natural habitats
Source: PLoS Biol. 2025 Jul 22;23(7):e3003285. doi: 10.1371/journal.pbio.3003285 (PMC12282910; doi:10.1371/journal.pbio.3003285)
Supplement: S13 Table — (DOCX) [file pbio.3003285.s018.docx]

**S13 Table: Variance inflation factors for the explanatory variables in abundance model**

| **Variable** | **VIF** |
| --- | --- |
| NDRS | 1.375441 |
| pnhRS | 1.291705 |
| crpRS | 1.354750 |
| tmpRS | 1.016780 |
| Predominant_land_use | 2.146364 |
| NDRS:crpRS | 1.519375 |
| NDRS:Predominant_land_use | 3.131734 |
| NDRS:Predominant_land_use:pnhRS | 3.919698 |

**Model formula:** logAbun ~ Predominant_land_use+pnhRS+ NDRS:crpRS+ NDRS:Predominant_land_use+ NDRS:Predominant_land_use:pnhRS+NDRS+tmpRS+crpRS + (1|SS) + (1|SSB)
