# Supplementary material for: Arthropod biodiversity loss from nitrogen deposition is buffered by natural and semi-natural habitats
Source: PLoS Biol. 2025 Jul 22;23(7):e3003285. doi: 10.1371/journal.pbio.3003285 (PMC12282910; doi:10.1371/journal.pbio.3003285)
Supplement: S1 Text — (DOCX) [file pbio.3003285.s019.docx]

**S1 Text: Model structure and selection**

To address our hypotheses, we built the minimum adequate mixed-effect model associated with N deposition for species richness and total abundance respectively by backward stepwise selection. Where, “NDRS” means nitrogen deposition after standardized, “tmpRS” represents mean annual temperature after standardized, “preRS” represents mean annual precipitation after standardized, “NFRS” means nitrogen fertilizer application after standardized, “phRS” means soil pH after standardized, “Predominant_land_use” means land use types of each sampling site, “pnhRS” means percentage of natural/semi-natural habitat of surrounding landscape after standardized, “cropRS” means percentage of cropland of surrounding landscape after standardized.

- Richness model <- GLMERSelect (modelData = model_data,

responseVar = "Species_richness",

fitFamily = "poisson",fixedFactors = c("Predominant_land_use"),

fixedTerms = list (NDRS,tmpRS,preRS,phRS, crpRS, NFRS),

randomStruct = "(1|SS) + (1|SSB) + (1|SSBS)",

fixedInteractions = c ("NDRS: tmpRS","NDRS: preRS",

" NDRS: phRS", " NDRS: pnhRS"," NDRS: crpRS",

" NDRS:Predominant_land_use",

" NDRS:Predominant_land_use: pnhRS"))

**Final species richness model:**

Species_richness ~ Predominant_land_use+pnhRS+ NDRS:tmpRS+ NDRS:crpRS+ NDRS:Predominant_land_use+ NDRS:Predominant_land_use:pnhRS+NDRS+tmpRS+crpRS + (1|SS) + (1|SSB) + (1|SSBS)

- Abundance model <- GLMERSelect (modelData = model_data,

responseVar = " logAbun",

fitFamily = "gaussian",fixedFactors = c("Predominant_land_use"),

fixedTerms= list (NDRS,tmpRS,preRS,phRS,pnhRS,crpRS, NFRS),

randomStruct = "(1|SS) + (1|SSB) ",

fixedInteractions = c ("NDRS: tmpRS","NDRS: preRS",

" NDRS: phRS", " NDRS: pnhRS"," NDRS: crpRS",

" NDRS:Predominant_land_use",

" NDRS:Predominant_land_use: pnhRS"))

**Final total abundance model:**

logAbun ~ Predominant_land_use+pnhRS+ NDRS:crpRS+ NDRS:Predominant_land_use+ NDRS:Predominant_land_use:pnhRS+NDRS+tmpRS+crpRS+ (1|SS) + (1|SSB
