# Supplementary material for: Arthropod biodiversity loss from nitrogen deposition is buffered by natural and semi-natural habitats
Source: PLoS Biol. 2025 Jul 22;23(7):e3003285. doi: 10.1371/journal.pbio.3003285 (PMC12282910; doi:10.1371/journal.pbio.3003285)
Supplement: S2 Text — (DOCX) [file pbio.3003285.s020.docx]

**S2 Text: Test of finer N deposition data with 0.1° resolution**

Data with finer spatial resolution would be helpful for more precise results when relating field sampled diversity data with larger scale N deposition. However, the current available global N deposition data has a coarse resolution of 1°×1° grid. To test whether this 1°×1°grid N deposition data is feasible to derive a sound result, we compared the results when two datasets of with different resolution were used. One N deposition data was derived from (HTAP II) project model as described in section 2.1.2 with the resolution of 1°×1°, and another was from the EMEP MSC-W model output of the European Monitoring and Evaluation Program for Long-Range Transboundary Transport of Air Pollutants (EMEP) with a data resolution of 0.1°×0.1° at the European scale (S2 Fig). Because of the high resolution of the European data, we excluded coordinates that do not belong to the European region and finally obtained data of 3480 sample sites. According to the description in the method section (see method), we extracted the N deposition data for 2010 for each sample site using the coordinates and standardized the N deposition data. To verify the correlation between EMEP and HTAP II data in order to compare the two data sets, we performed a Pearson correlation test on the two data sets with a p-value of 0.851, indicating a strong correlation between the data. We ran the same model to investigate the effects of N deposition on arthropod diversity in difference with the European N deposition data of finer resolution. The results of the two data sets both showed the negative effects of N deposition on arthropod species diversity vary across different land use, however this negative effect is stronger than when coarse resolution N deposition data was used (S9 Table, S10 Table). However, since the data only include Europe, this negative effect may be amplified and needs to be further verified with finer resolution global N deposition data thereafter.
