# Supplementary material for: Arthropod biodiversity loss from nitrogen deposition is buffered by natural and semi-natural habitats
Source: PLoS Biol. 2025 Jul 22;23(7):e3003285. doi: 10.1371/journal.pbio.3003285 (PMC12282910; doi:10.1371/journal.pbio.3003285)
Supplement: S3 Text — (DOCX) [file pbio.3003285.s021.docx]

**S3 Text: The effects of the total N deposition on species richness of Arthropods when Chao estimated species richness index was used**

Differences in sampling effort can bias estimates of species richness. Considering the sampling effort in the PREDICTS database is recorded using very different measures and units. To avoid any differences that we are unable to account for in the main analysis. From our selected dataset, only 4,781 sites can be used to calculated Chao-estimated species richness. We run the model selected by backward stepwise selection for species richness, using Chao-estimated species richness as the response variable instead of observed species richness. The patterns of the results were broadly similar to those presented in the main text. Across all land uses, biodiversity in Pasture showed the strongest decline with increasing N deposition, following by Primary vegetation, Secondary vegetation and Cropland (S3 Fig, S11 Table). But biodiversity in Plantation forest showed a different trend. However, given the limited number of sampling sites, the positive effects of N deposition in Plantation forest need to be validated with more field observations in the future.
