# Supplementary material for: Arthropod biodiversity loss from nitrogen deposition is buffered by natural and semi-natural habitats
Source: PLoS Biol. 2025 Jul 22;23(7):e3003285. doi: 10.1371/journal.pbio.3003285 (PMC12282910; doi:10.1371/journal.pbio.3003285)
Supplement: Attachment — Submitted filename: PBIOLOGY-D-25-00837_R1_reviewer3.pdf [file pbio.3003285.s023.pdf]

PLOS Biology

Landscape composition mediates global losses in arthropod diversity caused by atmospheric nitrogen deposition

--Manuscript Draft--

**Manuscript Number:**	PBIOLOGY-D-25-00837R1
**Full Title:**	Landscape composition mediates global losses in arthropod diversity caused by atmospheric nitrogen deposition
**Short Title:**	Landscape mediate losses in arthropod biodiversity caused by N deposition
**Article Type:**	Short Reports
**Keywords:**	biodiversity, semi-natural and natural habitat, landscape, buffer effect, land use type
**Abstract:**	Nitrogen (N) deposition is known to strongly modify biogeochemical cycles and trophic interactions, in turn altering ecosystem functioning and plant diversity around the globe. However, our understanding of N deposition effects on arthropod diversity remains limited. Here, we investigate how N deposition impacts the diversity of arthropods by combining biodiversity data from the PREDICTS database with data on global N deposition and land cover using mixed-effects models. We then explore the potential for semi-natural and natural habitats ('SNH') to buffer potential N deposition-linked biodiversity losses. N deposition has a negative effect on arthropod biodiversity. Both, species richness and abundance are significantly reduced in areas of high observed N deposition when compared to areas experiencing low N deposition, with responses varying for different land-use types. The strongest negative effects of N deposition on arthropod diversity were observed in landscapes experiencing the least anthropogenic modifications. With the exception of cropland-dominated landscapes, increases in SNH proportions reduced N deposition-induced arthropod biodiversity losses. We conclude that SNH can play an important role in mitigating negative effects of N deposition on arthropod diversity, with the conservation and creation of these habitats promoting arthropod diversity even under high N deposition levels.
**Additional Information:**	
**Question**	**Response**
**Financial Disclosure**<br><br>Enter a financial disclosure statement that describes the sources of funding for the work included in this submission. Review the submission guidelines for detailed requirements. View published research articles from *PLOS Biology* for specific examples.<br><br>This statement is required for submission and **will appear in the published article** if the submission is accepted. Please make sure it is accurate.	Yes

Powered by Editorial Manager® and ProduXion Manager® from Aries Systems Corporation

**Funded studies**<br>Enter a statement with the following details:<br>• Initials of the authors who received each award<br>• Grant numbers awarded to each author<br>• The full name of each funder<br>• URL of each funder website<br>• Did the sponsors or funders play any role in the study design, data collection and analysis, decision to publish, or preparation of the manuscript?<br><br>Did you receive funding for this work?	
Please add funding details.<br>as follow-up to "**Financial Disclosure**<br><br>Enter a financial disclosure statement that describes the sources of funding for the work included in this submission. Review the submission guidelines for detailed requirements. View published research articles from *PLOS Biology* for specific examples.<br><br>This statement is required for submission and **will appear in the published article** if the submission is accepted. Please make sure it is accurate.<br><br>**Funded studies**<br>Enter a statement with the following details:<br>• Initials of the authors who received each award<br>• Grant numbers awarded to each author<br>• The full name of each funder<br>• URL of each funder website<br>• Did the sponsors or funders play any role in the study design, data collection and analysis, decision to publish, or preparation of the manuscript?<br><br>Did you receive funding for this work?"	This work was supported by National Natural Science Foundation of China (no.41871186). S.F. was also supported by the China Scholarship Council (202306350102) to study and work with T.N. at CBER, University College London. T.N. was supported by a grant from the Natural Environment Research Council (NE/R010811/1) and by a University Research Fellowship from the Royal Society.
Please select the country of your main research funder (please select carefully as in some cases this is used in fee calculation).	CHINA - CN

as follow-up to "**Financial Disclosure**<br><br>Enter a financial disclosure statement that describes the sources of funding for the work included in this submission. Review the submission guidelines for detailed requirements. View published research articles from *PLOS Biology* for specific examples.<br><br>This statement is required for submission and **will appear in the published article** if the submission is accepted. Please make sure it is accurate.<br><br>**Funded studies**<br>Enter a statement with the following details:<br>• Initials of the authors who received each award<br>• Grant numbers awarded to each author<br>• The full name of each funder<br>• URL of each funder website<br>• Did the sponsors or funders play any role in the study design, data collection and analysis, decision to publish, or preparation of the manuscript?<br><br>Did you receive funding for this work?"	
**Competing Interests**<br><br>You are responsible for recognizing and disclosing on behalf of all authors any competing interest that could be perceived to bias their work, acknowledging all financial support and any other relevant financial or non-financial competing interests.<br><br>Do any authors of this manuscript have competing interests (as described in the PLOS Policy on Declaration and Evaluation of Competing Interests)?	The authors have declared that no competing interests exist.

Powered by Editorial Manager® and ProduXion Manager® from Aries Systems Corporation

**If yes**, please provide details about any and all competing interests in the box below. Your response should begin with this statement: *I have read the journal's policy and the authors of this manuscript have the following competing interests:*<br><br>**If no** authors have any competing interests to declare, please enter this statement in the box: "*The authors have declared that no competing interests exist.*"<br><br>* typeset<br><br>This statement is required for submission and **will appear in the published article** if the submission is accepted. Please make sure it is accurate and that any funding sources listed in your Funding Information later in the submission form are also declared in your Financial Disclosure statement.	
**Ethics Statement**<br><br>You must include an Ethics Statement in the Methods section of your manuscript if your study involved:<br>• Human participants<br>• Human specimens or tissue<br>• Vertebrate animals or cephalopods<br>• Vertebrate embryos or tissues<br>• Field research<br><br>**Not including a statement when required will delay the review process.**<br><br>General guidance is provided below. Please consult the guidelines on human subjects research and animal research for detailed instructions.	I confirm that I have read and understood PLOS' requirements for an Ethics Statement.

**Format for specific study types**<br><br>**Human Subject Research (involving human participants and/or tissue)**<br>• Give the name of the institutional review board or ethics committee that approved the study<br>• Include the approval number and/or a statement indicating approval of this research<br>• Indicate the form of consent obtained (written/oral) or the reason that consent was not obtained (e.g. the data were analyzed anonymously)<br><br>**Animal Research (involving vertebrate animals, embryos or tissues)**<br>• Provide the name of the Institutional Animal Care and Use Committee (IACUC) or other relevant ethics board that reviewed the study protocol, and indicate whether they approved this research or granted a formal waiver of ethical approval<br>• Include an approval number if one was obtained<br>• If the study involved *non-human primates*, add *additional details* about animal welfare and steps taken to ameliorate suffering<br><br>**Field Research**<br><br>Include the following details if this study involves the collection of plant, animal, or other materials from a natural setting:<br>• Field permit number<br>• Name of the institution or relevant body that granted permission<br><br>Please check the box to confirm your understanding of this policy, then read and agree to one of the statements.	
Please respond:<br>as follow-up to "**Ethics Statement**<br><br>You must include an Ethics Statement in	My study does not require an ethics statement.

Powered by Editorial Manager® and ProduXion Manager® from Aries Systems Corporation

the Methods section of your manuscript if your study involved:<br>• Human participants<br>• Human specimens or tissue<br>• Vertebrate animals or cephalopods<br>• Vertebrate embryos or tissues<br>• Field research<br><br>**Not including a statement when required will delay the review process.**<br><br>General guidance is provided below. Please consult the guidelines on human subjects research and animal research for detailed instructions.	

**Format for specific study types**<br><br>**Human Subject Research (involving human participants and/or tissue)**<br>• Give the name of the institutional review board or ethics committee that approved the study<br>• Include the approval number and/or a statement indicating approval of this research<br>• Indicate the form of consent obtained (written/oral) or the reason that consent was not obtained (e.g. the data were analyzed anonymously)<br><br>**Animal Research (involving vertebrate animals, embryos or tissues)**<br>• Provide the name of the Institutional Animal Care and Use Committee (IACUC) or other relevant ethics board that reviewed the study protocol, and indicate whether they approved this research or granted a formal waiver of ethical approval<br>• Include an approval number if one was obtained<br>• If the study involved *non-human primates*, add *additional details* about animal welfare and steps taken to ameliorate suffering<br><br>**Field Research**<br><br>Include the following details if this study involves the collection of plant, animal, or other materials from a natural setting:<br>• Field permit number<br>• Name of the institution or relevant body that granted permission<br><br>Please check the box to confirm your understanding of this policy, then read and agree to one of the statements. "	
**Data Availability**<br><br>PLOS journals require authors to make all data underlying the findings described in their manuscript fully available, without restriction and from the time of	Yes - all data are fully available without restriction

publication, with only rare exceptions to address legal and ethical concerns (see the PLOS Data Policy and FAQ for further details). When submitting a manuscript, authors must provide a Data Availability Statement that describes where the data underlying their manuscript can be found.<br><br>Your answers to the following constitute your statement about data availability and will be included with the article in the event of publication. **Please note that simply stating 'data available on request from the author' is not acceptable.** ***If, however, your data are only available upon request from the author(s), you must answer "No" to the first question below, and explain your exceptional situation in the text box provided.***<br><br>Do the authors confirm that all data underlying the findings described in their manuscript are fully available without restriction?	
Please describe where your data may be found, writing in full sentences. **Your answers should be entered into the box below and will be published in the form you provide them, if your manuscript is accepted.** If you are copying our sample text below, please ensure you replace any instances of XXX with the appropriate details.<br><br>• If your data are all contained within the paper and/or Supporting Information files, please state this in your answer below. For example, "All relevant data are within the paper and its Supporting Information files."<br>• If your data are held or will be held in a public repository, include URLs, accession numbers or DOIs. For example, "All XXX files are available from the XXX database (accession	The data that support the findings of this study are openly available in github at https://github.com/fanshunxiang/arthropod

Powered by Editorial Manager® and ProduXion Manager® from Aries Systems Corporation

number(s) XXX, XXX)." If this information will only be available after acceptance, please indicate this by ticking the box below.<br>• If neither of these applies but you are able to provide details of access elsewhere, with or without limitations, please do so in the box below. For example:<br><br>"Data are available from the XXX Institutional Data Access / Ethics Committee for researchers who meet the criteria for access to confidential data."<br><br>"Data are from the XXX study whose authors may be contacted at XXX."<br><br>* typeset	
Additional data availability information:	Tick here if the URLs/accession numbers/DOIs will be available only after acceptance of the manuscript for publication so that we can ensure their inclusion before publication.

Powered by Editorial Manager® and ProduXion Manager® from Aries Systems Corporation

Manuscript Click here to access/download;Manuscript;Manuscript.docx

Landscape composition mediates global losses in arthropod diversity caused by atmospheric nitrogen deposition

Shunxiang Fan[a], Tim Newbold[b], Jan C Axmacher[c], Charlotte L. Outhwaite[bd], Yi Zou[e], Zhenrong Yu[a], Yunhui Liu[a]*

a Beijing Key Laboratory of Biodiversity and Organic Farming, College of Resources and Environmental Sciences, China Agricultural University, Beijing 100193, China.

b Centre for Biodiversity and Environment Research, Department of Genetics, Evolution & Environment, University College London, London, WC1E 6BT, United Kingdom.

c Department of Geography, University College London, London, WC1E 6BT, United Kingdom.

d Institute of Zoology, Zoological Society of London, Outer Circle, Regent's Park, London, United Kingdom.

e Department of Health and Environmental Sciences, School of Science, Xi'an Jiaotong-Liverpool University, Suzhou, China

* Corresponding author at: College of Resources and Environmental Sciences, China Agricultural University, 2# West Yuan Ming Yuan Road, Beijing 100193, China.

Abstract: Nitrogen (N) deposition is known to strongly modify biogeochemical cycles and trophic interactions, in turn altering ecosystem functioning and plant diversity around the globe. However, our understanding of N deposition effects on arthropod diversity remains limited. Here, we investigate how N deposition impacts the diversity of arthropods by combining biodiversity data from the PREDICTS database with data on global N deposition and land cover using mixed-effects models. We then explore the potential for semi-natural and natural habitats ('SNH') to buffer potential N deposition-linked biodiversity losses. N deposition has a negative effect on arthropod biodiversity. Both, species richness and abundance are significantly reduced in areas of high observed N deposition when compared to areas experiencing low N deposition, with responses varying for different land-use types. The strongest negative effects of N deposition on arthropod diversity were observed in landscapes experiencing the least anthropogenic modifications. With the exception of cropland-dominated landscapes, increases in SNH proportions reduced N deposition-induced arthropod biodiversity losses. We conclude that SNH can play an important role in

mitigating negative effects of N deposition on arthropod diversity, with the conservation and creation of these habitats promoting arthropod diversity even under high N deposition levels.
Key words: biodiversity, semi-natural and natural habitat, landscape, buffer effect, land use type

Introduction

Nitrogen (N) is an essential nutrient for plant growth and productivity, and its availability strongly affects ecosystem functioning by influencing interspecific competition and trophic relationships (Kuypers et al., 2018; Sutton et al., 2011; Vitousek et al., 1997). Naturally, atmospheric N deposition (N_{dep}) (Galloway et al., 2008) and microbial N fixation (Kuypers et al., 2018) form key N sources in terrestrial ecosystems. Human activities, notably the burning of fossil fuels and chemical fertilizer applications have at least doubled total average N deposition (hereafter referred to as atmospheric N deposition) rates in less than a century (Schlesinger, 2009). Current N deposition rates have been proposed as a major threat to global terrestrial biodiversity (Jaureguiberry et al., 2022; Payne et al., 2017; Sage, 2020). Nonetheless, while large-scale impacts of land use and climate change on the biodiversity of many animal taxa are increasingly recognized and understood (e.g. (Ganuza et al., 2022; Neff et al., 2022; Newbold et al., 2015; Outhwaite, McCann, et al., 2022; Semenchuk et al., 2022; Soroye et al., 2020)), global-scale effects of N deposition require greater attention. Global N deposition levels continue to increase, despite ongoing efforts to reduce them (Ackerman et al., 2019; Hicks et al., 2011; Wen et al., 2020), implying that any associated potential threats to biodiversity are likely to increase, too. A better understanding of the nature and strength of N deposition impacts on the diversity especially of the world's most species-rich taxa are therefore critical to inform effective global biodiversity conservation planning.

Previous studies on the impacts of N deposition on biodiversity have mostly focused on plant species and local scales. Relying strongly on data from field N deposition experiments, they show that high N deposition rates trigger losses in plant diversity and changes in vegetation composition (Bebber, 2021; Roth et al., 2015; Schipper et al., 2020; Simkin et al., 2016; Stevens, Duprè, et al., 2010; Zhang et al., 2023). While research into responses of animal assemblages to N deposition have remained limited (Nijssen et al., 2017; Stevens et al., 2018; WallisDeVries & Bobbink, 2017), it might be expected that plant community responses translate up to higher

trophic levels. Increasing plant productivity might provide greater food resources at higher trophic levels, potentially allowing a greater range of herbivorous species to exist in an N-rich environment (Luff, 1966; Woodcock et al., 2007), while, alternatively, an associated decrease in plant diversity might limit feeding niche diversity across trophic levels (Le Provost et al., 2020), hence resulting in parallel negative diversity responses across trophic levels in response to increased N deposition (Bobbink, Braun, et al., 2010; Pardo et al., 2011). N deposition rates are furthermore impacted by land use and landscape patterns. For example, the conversion of agricultural land to semi-natural forest or grassland ecosystems often reduces regional N deposition rates on remaining agricultural land due to changing ground surface features (surface roughness and soil moisture levels), which are important to dry and wet N deposition (Lu et al., 2016). Nonetheless, large-scale responses of animal biodiversity to N deposition under different land use types remains poorly understood.

Arthropods that represent the most species-rich taxa of terrestrial animals, play critical roles in ecosystem functions, ranging from pollination, decomposition and nutrient cycling, pest control, soil aeration and fertility to seed dispersal (Chakravarthy et al., 2016). A growing body of literature indicates a continued decline in their diversity (Ghisbain et al., 2024; Nicholson et al., 2024; Outhwaite et al., 2022) with cascading effects on ecosystems and, ultimately, human well-being (Chakravarthy et al., 2016). To date, the impact of N addition on arthropods have only been studied at highly localized scales. In this context, elevated N levels have been indicated to heighten risks of pest outbreaks through enhanced foliar palatability (Maskell et al., 2010), while negatively impacting pollinating insects by altering the chemical makeup of nectar and pollen (Ceulemans et al., 2017; Stevens, Thompson, et al., 2010), indirectly resulting in a decline in pollination services and crop yields (Klein et al., 2007).

On a landscape scale, atmospheric N deposition may increase particularly in cropland-dominated landscapes, with crop management linked to key additional sources of N deposition (L. Liu et al., 2022) that will affect biodiversity also in surrounding areas (Dragosits et al., 2006). Understanding N deposition impacts on arthropod communities on a global scale can in this context provide critical new insights

for safeguarding associated ecosystem services particularly within agricultural landscapes.

In addition to N deposition, the transformation of semi-natural and natural land for agricultural use has been widely recognized as a core driver of global biodiversity los[illegible]n agricultural landscapes, semi-natural and natural habitats (hereafter SNH) are important for the maintenance of biodiversity and associated ecosystem services (Mestre et al., 2018; Outhwaite, Ortiz, et al., 2022; Papanikolaou et al., 2017). Their presence is already known to mitigate adverse effects of increasing temperature (Outhwaite, McCann, et al., 2022; Papanikolaou et al., 2017) and intensive agricultural production (Tscharntke et al., 2012, 2021) on arthropod assemblages. SNH are believed to provide general refuge spaces for species under pressure from intensive cultivation practices, including from high N fertilizer applications on adjacent agricultural land. Consequen[illegible] SNH are expected to buffer detrimental effects of N deposition on agro-biodiversity. However, the expansion of agricultural land in response to increases in the human population and changing consumption patterns has led to an ongoing loss of SNH and increases in cropland areas, while also resulting in increased N deposition rates (Gallego-Zamorano et al., 2022). Such changes may disproportionately contribute to a sustained decline in species diversity (Gallego-Zamorano et al., 2022; Habel et al., 2019; Kormann et al., 2015), thereby posing a threat to plant and animal taxa challenging our ability to reach global biodiversity conservation goals (CBD, 2020). It is therefore essential to explore if and how SNHs buffer the negative effects of N deposition (from now on called “buffer effect”).

To explore this question, we combined data on global N deposition and the composition of arthropod assemblages across different land-use types to assess the relationship between N deposition and biodiversity (measured as species richness, with total abundance of arthropod assemblages also considered). Specifically, we extracted arthropod diversity data for 6,416 sites (including site-level land use information) from the PREDICTS database (Fig. 1). Using the coordinates of each site, we then extracted data on N deposition, proportion of semi-natural/natural habitats, proportion of cropland, and other covariates (temperature, precipitation, Soil PH, N fertilizer). Based

on this information, we conducted analyses using mixed-effects models to answer two core sets of questions: 1) Differentiated by land-use type, how does N deposition impact arthropod species richness and total abundance? Does the deposition of reduced N (ammonia) in this context exert a greater influence on arthropods than that of oxidized N (e.g. nitrous oxide)? 2) Can the presence and extent of SNH in the surrounding landscape buffer detrimental effects of N deposition on arthropod diversity, particularly within cropland sites? We hypothesize that (1) With the increase in N deposition, arthropod diversity generally declines irrespective of land-use type. Deposition of reduced N furthermore has the most negative effect, not least due to the associated acidification of soils. (2) SNH can buffer deposition effects on biodiversity.

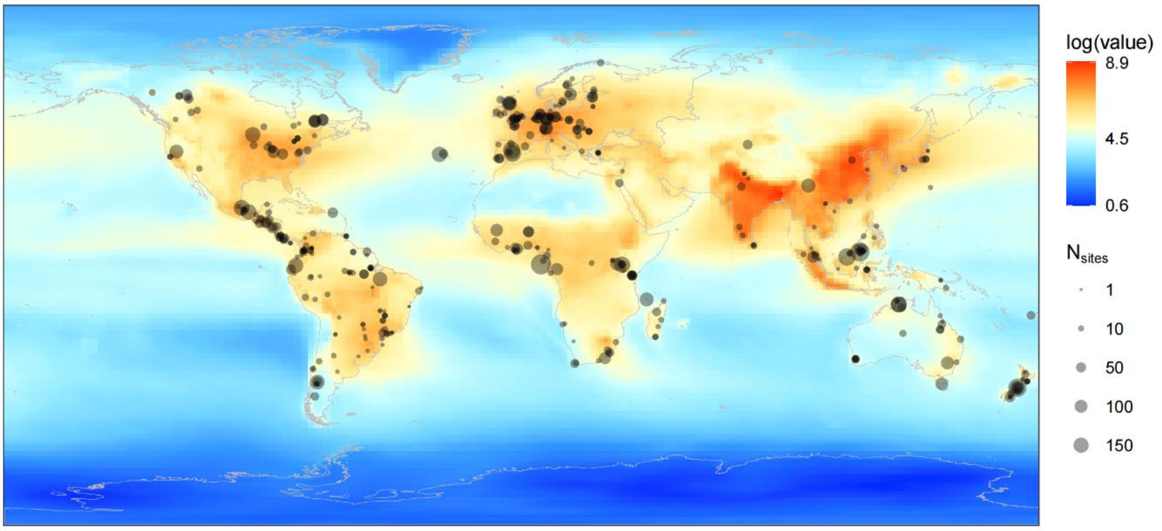

Fig. 1: The distribution of sites from the PREDICTS database and the level of total N deposition. The size of the point represents the number of sites per study (N_{sites}). The change from blue to orange indicates the increase in the total amount of atmospheric N deposition. N deposition values were $\log_e$-transformed, with N deposition ranging from 0.8 mg N m^{-2} yr^{-1} to 7960.0 mg N m^{-2} yr^{-1}.

Methods

Arthropod biodiversity and land use data

Data on the composition of arthropod assemblages across different land-use types were extracted from the PREDICTS database (Hudson et al., 2016, 2023). This global terrestrial biodiversity database contains spatial comparisons of local biodiversity from sites of differing land use and/or land-use intensity. The complete dataset includes 480 data sources (published articles, or unpublished datasets, but with published methodology) with ~3.2 million species records, covering all major land regions of the world except the Antarctica. The temporal extent of the database covers the years 1984 to 2013, with most data concentrated after 2000 (Hudson et al., 2017). The database has a hierarchical structure, with each data source containing one or more 'studies', where data were collected using the same method (666 studies in total), each study containing one or more spatial 'blocks', representing distinct spatial clusters of sites, and each block containing a number of specific sampling 'sites'. Each site has records of species abundance or occurrence, from which it is possible to derive measures of community composition, and each study site is also assigned a standardized land-use type (see below for details) (Hudson et al., 2014, 2016, 2017). Almost all sampled sites within PREDICTS are accompanied by geographical coordinates giving their precise location.

In our study, we selected data for Arthropods species in the PREDICTS database, including 6 taxonomic classes: Arachnida, Chilopoda, Diplopoda, Entognatha, Insecta, and Malacostraca, which together accounted for 1,121,105 records. We calculated two site-level measures to use as response variables in our models: total abundance (total number of individuals across all species sampled at a site) and species richness (the total number of species – as defined by the original study authors – sampled at a site). If the sampling effort varied across sites within a study, sampling effort was scaled so that the highest value of sampling effort was 1 and all others were relative to that. The abundance measurements were then divided by this relative sampling effort. We therefore assume that there is a linear relationship between recorded abundance and sampling effort (Newbold et al., 2014). Sites with the same coordinates and land-use

conditions were merged to combine sub-samples from the same location (e.g., multiple pitfall traps in an array of traps). As sample sizes are incomplete and vary, we also calculated the Chao-1 estimator (Chao et al., 2005). While being aware of the limitations of species estimators in comparing incompletely sampled communities, we employed the Chao-1 estimator as a surrogate to verify the consistency of our results with the observed species richness. The patterns of the results were broadly similar to those presented in the main text (the results of the models based on Chao-estimated richness are presented in Supplementary 4).

The PREDICTS database assigns sites to specific land-use classes based on information provided in the source publication or by enquiry to the original study authors. Land uses are divided into six categories: (1) Primary vegetation (PV), comprising forest or non-forested natural habitat that is not known to have been destroyed in the past; (2) Secondary vegetation (SV), which represents natural habitats that have been destroyed by human activities or extreme natural events in the past, but where vegetation is being restored or naturally recovering; (3) Cropland (CR), representing land used to grow herbaceous crops; (4) Plantation forest (PF), which represents areas used to grow timber or crop trees or shrubs for commercial or subsistence use; (5) Pasture (PA), comprising land known to be regularly or permanently grazed by livestock, and (6) Urban (UR), comprising areas covered with human habitation, buildings, or areas managed for recreation (e.g., parks). To study the effect of SNH on biodiversity responses to N deposition in the surrounding area, we focus on areas that are not dominated by impervious surfaces, thus excluding urban areas. We also excluded records with undetermined land use, and records missing either geographic coordinates or abundance data. The final dataset included total abundance and species richness data from 6,416 sampled sites, representing 270 studies.

Nitrogen deposition datasets

We selected and used outputs of the following 11 global deposition models from the second-phase mission of the Hemispheric Transport of Air Pollution (HTAP II) project(Galmarini et al., 2017; Tan et al., 2018) to obtain estimates of N deposition,

following the criteria of Tan et al.(Tan et al., 2018): CAM-Chem, C-IFS_v2, SPRINTARS, GOCARTv5, OsloCTM3v.2, GEOSCHEMAJOINT, GEOS5, EMEP_rv48, GEM-MACH, CHASER_re1 and CHASER_t106 re1 (Detail in Tan et al.(Tan et al., 2018); Schwede et al.(Schwede et al., 2018)). These 11 models were selected as they satisfied the following two criteria suggested by Tan et al.(Tan et al., 2018): (1) The modeled N deposition values range of the 11 models were limited within ± 20% of the N emission values (emission inventory grid are provided by seven different sectors, e.g. energy, industry); (2) Compared with the mean of all modelling deposition values, the modelled values of the these 11 models did not exceed ± 1.5 times the interquartile range of the multi-model average values (See further details in Tan et al.(Tan et al., 2018)). These outputs of N deposition were interpolated onto a 1° × 1° grid (resolution of ~111 km at the equator) by taking the arithmetic mean of values from these 11 models (Fig. 1). The dataset shows strong consistency with N deposition data from other datasets and observation networks (L. Liu et al., 2022; Schwede et al., 2018; Tan et al., 2018). The dataset provides two types of N deposition data: oxidized nitrogen forms (NO_Y, primarily generated from high-temperature processes including industrial processes and fossil-fuel combustion); and reduced nitrogen forms (NH_x, primarily originating from agricultural fertilizers and animal manure). The total N deposition was obtained by summing the estimates of oxidized N deposition and of reduced N deposition. Values of total oxidized and reduced N deposition for each sampling site were then extracted based on geographic coordinates. As previous studies have shown that the proportion of these two types of N deposition may change over time (Li et al., 2016), and the biological and ecological effects they produce may also differ (Schwede et al., 2018), we additionally compared the effects on arthropod biodiversity of the two types of N deposition separately (results are presented in Supplementary S2). Although data with a higher spatial resolution would be helpful for more precise results when relating field-sampled biodiversity to N deposition, global estimates of N deposition are not currently available at a spatial resolution finer than 1°. We checked the consistency of our results by running models for Europe, for which 0.1° × 0.1° estimates of N deposition are available. The results using the fine-resolution

N deposition data were consistent with results using the coarse N deposition data (see Supplementary S3 for more details).

Other variables potentially associated with N deposition impacts

In order to enhance the model's explanatory ability, we also considered additional factors (climate ~temperature and precipitation, soil PH, N fertilizer application) that may potentially influence the interaction between biodiversity and N deposition in our study (Borer & Stevens, 2022; Boutin et al., 2017; Cheng et al., 2019; L. Liu et al., 2022; Simkin et al., 2016). For climate data, we extracted estimates of mean annual temperature and mean annual precipitation for 2010 from CRU TS version 4.07(Harris et al., 2020) at 0.5° spatial resolution. The CRU TS dataset is a global meteorological dataset for all land regions except Antarctica, derived from interpolated monthly climate anomalies based on extensive networks of weather-station observations. It covers the period from 1901 to the present day, with an annual timestep. To obtain data on N fertilizer application, we utilized the Global Fertilizer and Manure database, Version 1, which combines national levels of crop fertilizer usage data obtained from the International Fertilizer Industry Association (IFA) with global harvested areas of 175 crops at a resolution of 0.5 degrees (Potter et al., 2010). It should be noted worth mentioning that N deposition data are associated with, and partly generated from, N fertilizer application data in the agriculture sector (Janssens-Maenhout et al., 2015; L. Liu et al., 2022), so the level of N deposition/fertilizer is also spatially correlated (Kleijn et al., 2009) and can serve as an indicator of human activity intensity. We compared N fertilizer data with N deposition data in model selection to assess their significant effects on biodiversity in this study. Soil PH data was extracted from SoilGrids250m version 2.0 (Hengl et al., 2017). It models the spatial distribution of global soil properties using global soil profile information and covariate data by machine learning to generate a collection of world soil attribute maps with a resolution of 250 meters. The four variables were extracted based on the geographic coordinates of each sample site.

Proportion of SNH and cropland in the surrounding landscape

We used global fine-scale estimates of land use for 2005 (Hoskins et al., 2016) to extract the proportion of SNH surrounding each sampling site. We selected the 2005 data as it was closest to the median sampling year of the PREDICTS sites included in our analysis. For the land-use dataset, we divided the terrestrial area into five types, corresponding with the classification used in PREDICTS − primary vegetation, secondary vegetation, pasture, cropland and urban. The dataset was created by statistically downscaling mapped coarse-scale land-use data (the 0.5° Land-use Harmonization data (Hurtt et al., 2011)) based on relationships with climate, topography, soil, land cover, population density, and accessibility. The downscaled dataset provides global mapped estimates of land use at a 30-arc-minute spatial resolution (approximately 1 km × 1 km at the equator) (Hoskins et al., 2016). Primary and secondary vegetation were grouped together as semi-natural and natural habitats (SNH). To estimate the proportion of SNH in the landscape surrounding each study site, we created a 5-km circular buffer around the midpoint of each sampling location and calculated the mean percentage combined coverage of primary and secondary vegetation within the buffer. The proportion of cropland was also extracted using the same method and dataset.

Statistical Analysis

We used mixed-effects models to explore the impact of N deposition on arthropod total abundance and species richness. N deposition (total) was treated as a one of fixed effects, either combined or separated by oxidized or reduced forms. Continuous variables, including N deposition, mean annual temperature, mean annual precipitation, soil N content, N fertilizer application, percentage of natural habitat, and percentage of cropland in the surrounding landscape were standardized using z-transformation to convert these into standardized, dimensionless values and were included as part of fixed effects as well. Study identity, and spatial block nested within study, were included as random effects to account for the differences in sampling methods used among studies, and the spatial distribution of sites within studies, respectively (Newbold et al., 2015).

The total abundance data contained many non-integer values, and thus we used a natural logarithm-transformation prior to fitting the data with a linear mixed-effects model with normally distributed errors. For species richness, generalized linear mixed-effects models with Poisson-distributed errors were used. Additionally, we also incorporated a random intercept of site identity in species richness model to control for overdispersion (Rigby et al., 2008). For more detail information on the models' output, refer to Supplementary Tables S3-S4. To illustrate the relative magnitudes of biodiversity responses associated with increasing N deposition, we calculated the model-estimated percentage difference in both total abundance and species richness across the range of N deposition values at the sites included in the analyses. We set the 2.5th percentile value of total N depositions as the reference value, i.e. as the value representing the low N deposition in this study, and sampled 10,000 estimates based on the model variance-covariance matrix to predict differences in abundance and richness compared to this reference. Median values and 95% confidence intervals thus represent the percentage difference compared to this baseline condition. Since results were similar for the separate models of oxidized and reduced N deposition (see Supplementary S2), we only used the total N deposition for subsequent analyses investigating the interaction effect with land use, proportion of SNH and proportion of cropland (hereafter, N deposition therefore refers to total N deposition).

To address our hypotheses, we built the minimum adequate mixed-effect model associated with N deposition for species richness and total abundance respectively by backward stepwise selection. Initially, we tested the interaction terms based on the most complex fixed-effects structure model. Subsequently, we removed them before examining the main effects. If the interaction effects are significant, the main effects will be retained regardless of their importance (Supplementary S1). Specifically, we included N deposition as a fixed effect to explore its effect on arthropod species richness and total abundance. N fertilizer was also included as a fixed effect in model selection to test whether it significantly influences biodiversity. Besides, other factors and their interaction with N deposition, including mean annual temperature, mean annual precipitation, and soil PH, which have been reported to potentially influence

biodiversity as well (Borer & Stevens, 2022; Boutin et al., 2017; Cheng et al., 2019; L. Liu et al., 2022), were included as covariables in the model. Additionally, to further investigate variations in the impact of N deposition across different land-use types (In our study, land use was a 5 levels categorical variable ~Primary Vegetation, Secondary Vegetation, Cropland, Plantation Forest and Pasture), we incorporated N deposition and its interaction with each land-use type into our analysis. Further, N deposition and percentage of SNH, as well as the proportion of cropland were also included as interacting fixed effects in the model, respectively, to investigate potential interactions between N deposition and SNH/cropland within a 5-km radius around each site on arthropod species biodiversity. Finally, we also added a three-way interaction between N deposition, the proportion of SNH and land use, to further explore whether the buffering effect of the proportion of SNH vary across land use. Model formulae and model selection can be found in Supplementary S1. We used variance inflation factors (VIFs) to assess collinearity among predictor variables. VIFs were all lower than 10, indicating low collinearity between predictors (Zuur et al., 2009) (Supplementary Table S12, S13).

We checked all the fitted models to assess the extent to which they met the model assumptions. All models are validated by checking their residual distribution following suggestions from Zuur (Zuur et al., 2009), and we used Moran's I to test for study-level spatial autocorrelation in the residuals (Supplementary S5). All statistical analyses were conducted in R 4.03 (R Core Team, 2013), with the models run using the lme4 package version 1.1-30 (Bates et al., 2015), the extraction of continuous raster data using the raster package version 3.5-15 (Hijmans et al., 2013), and the predictsFunctions package version 1.0 for final model selection and PREDICTS data processing (Newbold, 2018).

Data sources

Datasets used in our study: The PREDICTS data can be downloaded at https://Data.nhm.ac.uk/dataset/The-2016-release-of-The-PREDICTS-database. The Nitrogen deposition data can be downloaded at https://thredds.met.no/thredds/catalog/data/EMEP/Articles_data/Schwede_etal_Ndep_

2018/catalog.html. The land-use dataset used in our study to extract the proportion of semi-natural and natural habitats (SNH) and cropland in surrounding landscape can be downloaded at https://doi.org/10.4225/08/56DCD9249B224. The climatic zones were differentiated according to the Köppen-Geiger climate classification system, which can be downloaded at https://www.gloh2o.org/koppen.The climate data (Mean annual temperate and mean annual precipitation) were extracted from CRU TS version 4.07 at https://crudata.uea.ac.uk/cru/data/hrg/index.htm. The nitrogen fertilizer application data (Global Fertilizer and Manure, Version 1) can be downloaded at https://sedac.ciesin.columbia.edu/data/set/ferman-v1-nitrogen-fertilizer-application/data-download. The soil nitrogen content (SoilGrids250m version 2.0) data can be downloaded at https://soilgrids.org/.

Results

Land use-specific effects of N deposition on arthropod diversity

Across all land uses, arthropod biodiversity showed a significant decline with increasing N deposition both in terms of species richness and total abundance (Fig. 2). N deposition exhibited a significant negative effect on arthropod species richness in primary vegetation (p = 0.009; Table S3). Specifically, species richness at high-N deposition sites was 39% lower than at low-N deposition sites within primary vegetation (Fig. 2a). Species richness was about 24% lower in cropland sites experiencing high total atmospheric N deposition (2326.3 mg N m^{-2} yr^{-1}, representing the 97.5th percentile value of total N deposition in cropland) than at sites experiencing low N deposition (as the 2.5th percentile value of total N deposition) (Fig. 2a). Diversity in secondary vegetation ($p < 0.001$, Table S3) and pasture ($p = 0.011$, Table S3) ranked second (Table S3). Negative responses to N deposition were observed to be weakest in plantation forest diversity ($p < 0.001$, Table S3, Fig. 2a). Total abundance also showed varied responses to N deposition among the different land-use types (Fig. 2b, Table S4), with greatest losses observed in pasture ($p < 0.001$, Table S4), as well as primary

vegetation and secondary vegetation (Fig. 2b, Table S4). The effect of N deposition on total abundance in sites of cropland (45.14%, $p = 0.024$, Table S4) and plantation forest ($p < 0.001$, Table S6) were significantly lower compared to primary vegetation.

Similar responses of biodiversity to land use and N deposition were also observed when reduced N deposition and oxidized N deposition were analyzed separately (Supplementary S2). However, reduced N deposition had a more strong impact on species diversity (for species richness model $x^2_{22,23} = 23.13$, $p < 0.001$; total abundance model $x^2_{21,22} = 16.71$, $p < 0.001$), while impacts from oxidized N deposition were weaker (for species richness model $x^2_{22,23} = 8.04$, $p = 0.004$; total abundance model $x^2_{21,22} = 10.10$, $p = 0.001$). For example, when evaluating the impacts of N deposition on arthropod species richness in cropland, reduced N deposition leads to a 12% greater decline compared to oxidized N deposition.

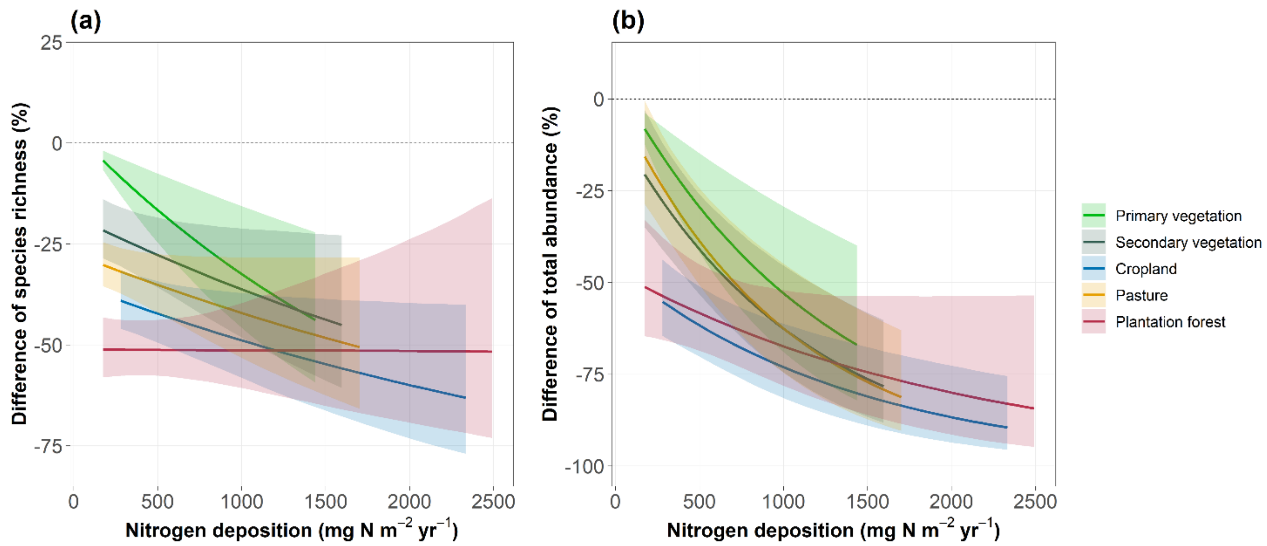

Fig. 2 The response of arthropod species richness (a) and total abundance (b) to the interaction of N deposition and land use. Likelihood ratio test of the interactive effect of N deposition and land use: species richness $x^2_{19,23} = 35.62$, $p < 0.001$; total abundance $x^2_{18,22} = 30.68$, $p < 0.001$. Values represent the percentage difference compared with primary vegetation sites with the 2.5th percentile value of total N deposition. The lines with different colors represent the median predicted value for each land-use type, with shaded areas representing the 95% confidence intervals. The results are predicted across 95% of the range of N deposition values for each land-use type. The number of sites were: Primary vegetation, $N_{site} = 1{,}615$; Secondary vegetation, $N_{site} = 1{,}511$; Cropland, $N_{site} = 1{,}563$; Pasture, $N_{site} = 1{,}347$; Plantation Forest, $N_{site} = 380$. The N deposition range used for plotting covers from 2.5% to 97.5% of sampled sites for all land-use types included in the model: 109.4 to 2491.6 mg N m^{-2} yr^{-1}.

The effect of SNH in buffering the impact of N deposition on arthropod diversity

Increases of N deposition had a less pronounced effect on species richness as SNH coverage in the landscape increased (Fig. 3). These SNH proportion-linked buffering effects were observed for all land-use types except cropland, while the strength of associated diversity benefits varied among land-use types (Fig. 3A). Buffering effects were stronger in primary vegetation, secondary vegetation than in pasture and planation forest (Fig. 3A a, b, d, e). While cropland biodiversity also generally benefits from a high percentage of SNH, although no buffering for the effects of N deposition was observed, as the declining trend in biodiversity was not affected by the increase in SNH. (Fig. 3A c). For total abundance, buffering effects were observed in pasture, plantation forest, secondary vegetation and primary vegetation (Fig. 3B a, b, d, e). Although there were no buffering effects observed for abundance patterns in cropland (Fig. 3B c). Although the declining trend in biodiversity was not affected by the increase in SNH, higher percentages of SNH still benefited biodiversity at least in sites with low levels of N deposition (N deposition lower around 1000 mg N m^{-2} yr^{-1}).

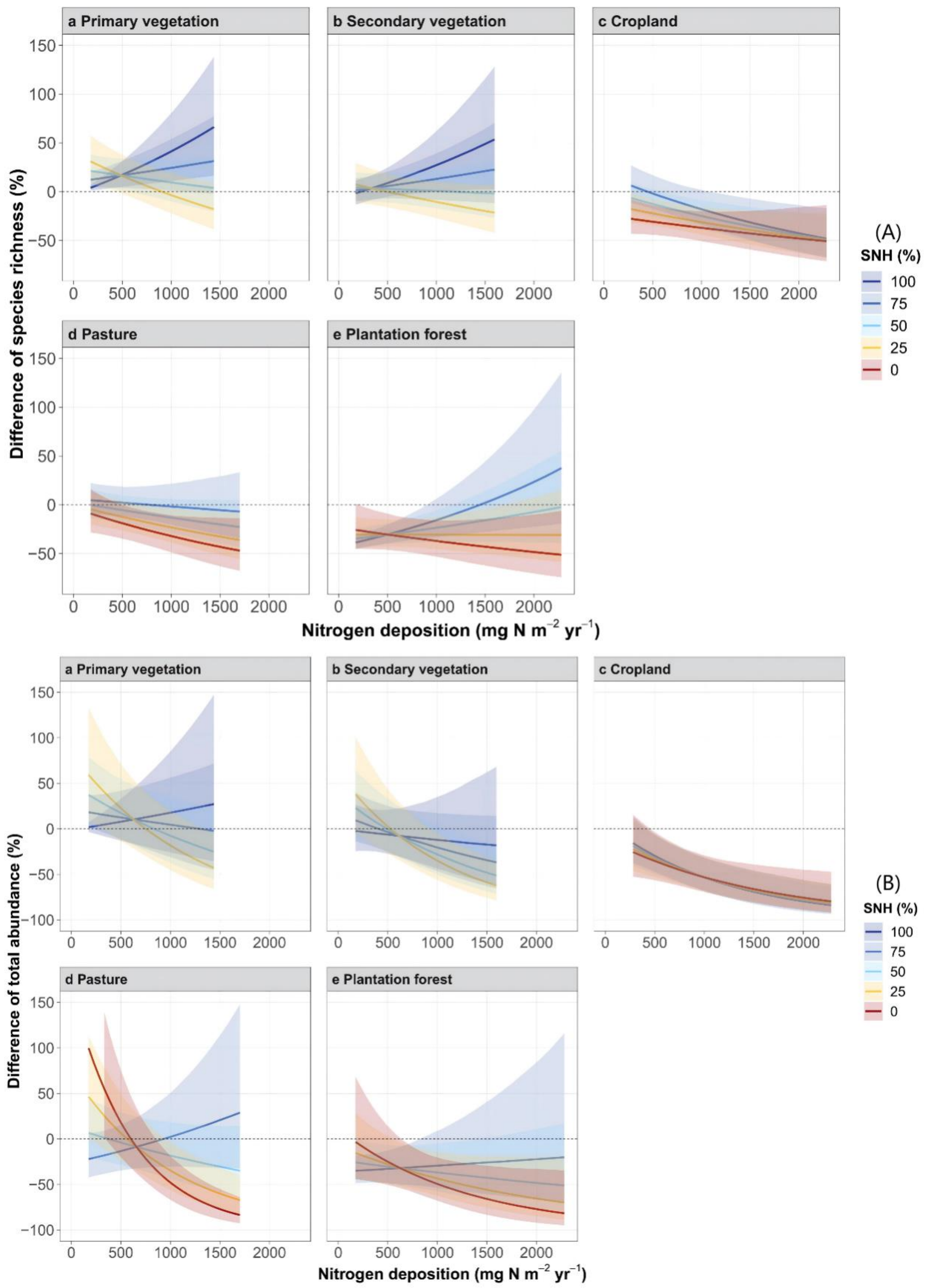

Fig. 3 The interactive effect of SNH and N deposition on arthropod species richness (A) and total abundance (B) in different land-use types. Likelihood ratio test of the interactive effect of N deposition and proportion of SNH in different land use types: species richness $x^2_{18,23}$ = 40.09, p = $p < 0.001$; total abundance $x^2_{17,22}$ = 33.81, $p < 0.001$. Values represent the percentage difference compared with primary vegetation with the 2.5th percentile value of total N deposition and 100% surrounding SNH. The lines with different colors represent the median predicted value of different percentages of SNH in the surrounding landscape, with shaded areas representing the 95%

confidence intervals.

Discussion

Earlier studies of the effects of N deposition on biodiversity mainly focused on plant diversity (Roth et al., 2015; Simkin et al., 2016; Stevens, Duprè, et al., 2010; Zhang et al., 2023). The few studies reporting the effects N deposition has on local animal biodiversity either focused on a small selection of taxa (Habel et al., 2016; Roth et al., 2021; Vogels et al., 2017; Wagner et al., 2021; WallisDeVries & Bobbink, 2017), or studied responses observed under controlled N deposition experiments data (Schipper et al., 2020). Here, we demonstrated, for the first time, a negative effect of N deposition on arthropod diversity at the global scale across different land use types. We furthermore highlighted the interactions between N deposition and landscape composition in influencing biodiversity, with particular focus on natural and semi-natural habitats.

Similar to previous studies on plant biodiversity, we found that deposition of reduced N, which is closely related to agricultural intensification, has a stronger negative impact on species diversity compared to oxidized N deposition (McCLEAN et al., 2011; Smart et al., 2004). This might most likely be linked to the aforementioned soil acidification uniquely linked to deposition of reduced forms of N (Midolo et al., 2019). Reduced (ammonium-dominated) N deposition will therefore alter soil microbial communities and plant secondary metabolites more severely, subsequently potentially disrupting arthropod trophic networks. A further increase in reduced N deposition in the future, as indicated by recent studies (Li et al., 2016; L. Liu et al., 2022), could trigger further direct losses in arthropod diversity. This highlights the importance of developing measures to reduce the negative impact of N deposition, and especially reduced N deposition, has on arthropod diversity (Li et al., 2016; L. Liu et al., 2022) Measures directly removing N from ecosystems, for example by targeted management like cutting and subsequent removal of biomass from grasslands, could be considered (Borer & Stevens, 2022; Jones et al., 2017). This may attenuate the negative effects of N deposition on species through biomass removal by grazers and the

offsetting of light limitations due to changes in the vegetation structure (Jones et al., 2017), or by altering species competition when creating open space and a mosaic of microhabitats that enhances biodiversity (Plassmann et al., 2010). It should be noted that these measures might also have negative side-effects, as such disturbances can directly kill specimens or temporarily reduce the amount of food available (Humbert et al., 2009). Additionally, appropriate agricultural strategies and livestock production measures should be implemented (Liu et al., 2022), via the expansion of extensive of highly N-efficient agricultural management approaches. In addition, since animal production usually coincides with high N emissions (per calory/unit protein produced), another effective measure might be to replace animal-based with plant-based products or replace vertebrate-based with arthropod-based protein, obviously requiring a substantial shift in diets.

Similar to earlier studies (Bobbink, Hicks, et al., 2010; Maskell et al., 2010; Matson et al., 2002; Pardo et al., 2011), we show that N deposition impacts on arthropod diversity are not acting uniformly across land-use types. Due to primary vegetation and secondary vegetation commonly being located far from major sources of N emissions, and historically experiencing low levels of N deposition (Bobbink, Hicks, et al., 2010) when compared for example to plantation forests or pastures, its biodiversity may show comparatively high sensitivity to N deposition. Meanwhile, as highlighted by Nijssen et al., (2017), an observed decline in animal diversity is frequently linked to structural changes in plant communities, reduced edibility of plant resources, and habitat modifications induced by vegetation shifts. In addition, N deposition also interacts with management practices; higher intensity land use is per se associated both with biodiversity losses and high levels of N exposure from deposition and direct application (Perring et al., 2018). Variations in soil properties, such as the degree of acidification, further influence species richness (Stevens et al., 2018). Therefore, the combined effects of these variables under different land-use types lead to a differential impacts of N deposition on biodiversity across varying land-use (Bobbink et al., 1998) (Fig 2).

The negative response of species richness to N deposition in cropland could be partly related to long-term exposure to N deposition, and to cropland soils becoming

increasingly acidic due to increasing N levels related to fertilization applications in combination with atmospheric deposition (Guo et al., 2010). Higher N deposition also leads to direct soil N enrichment and nitrification (Stevens et al., 2011; Sutton et al., 1998), which may result in increased risk of pest outbreaks (Maskell et al., 2010) that in turn require more intensive applications of pesticides that further affect biodiversity in anthropogenic habitats like cropland, but also plantation forests and pasture. As many species in landscapes with a high cropland cover will have been filtered out (Sirami et al., 2019), already, the species pool left at such sites may be highly stress-tolerant and pre-adapted to high levels of N exposure, resulting in limited diversity responses in such landscapes. In pasture, the increased prevalence of N-demanding species at the expense of less competitive ones may lead to a reduction in food resources, thereby negatively impacting the diversity of arthropod communities (Stevens et al., 2004). In Central Europe for example, grassland species richness decreased by approximately 23% with increasing N deposition (Stevens et al., 2004), a trend similar to our results for arthropod richness. N enrichment may also increase biomass accumulation in the vegetation that in turn changes the vegetation structure and could enhance fire risks during dry spells (Davidson et al., 2012), again potentially adversely affecting arthropod communities. It can be anticipated that changes in arthropod diversity within more natural habitats may primarily be driven by N deposition-induced shifts in plant community composition toward species less preferred by arthropods, or a homogenization and increased density of the vegetation providing a reduction in ecological niche space. In contrast, responses in human-dominated landscapes such as cropland will more likely be influenced by habitat alteration caused by, or associated with enhanced N deposition. However, these interpretations remain hypothetical and require further empirical validation through specific case studies (Nijssen et al., 2017).

Consistent with our hypothesis, increasing the amount of SNH in the surrounding landscape partly buffers adverse effects of N deposition on arthropod biodiversity. These positive buffering effects might in the case of forests coverage be related to their ability to directly reduce impacts of atmospheric N deposition on surrounding areas by removing N compounds in their complex canopies (Hicks et al., 2011). Meanwhile,

some managed woodlands for example in agricultural landscapes have not only a large leaf area, but create increased turbulence that helps dilute N deposition, thereby reducing the impact on the forest ground (Dragosits et al., 2006; Theobald et al., 2001), but also on surrounding agricultural land, with these SNH acting as N sinks (Deng et al., 2019; Hicks et al., 2011). Similarly, compared to areas without planted shrubs, areas with planted shrubs will experience less biodiversity loss from N deposition, as N will not accumulate directly in the soil, which can help to mitigate biodiversity loss (Shao et al., 2018). Furthermore, an increase in SNH generally increases the landscape heterogeneity which contributes positively towards conserving high species diversity at the landscape level by providing additional habitat niches, resources, and potential refuge sites (Mestre et al., 2018; Poggio et al., 2010; Rundlöf & Smith, 2006). Therefore, preserving or establishing SNH across agricultural landscapes (e.g. plantation, pasture, cropland) offers a potential mitigation measure to address some of the negative effects N deposition exerts on biodiversity.

Considering the ongoing global declines in arthropod populations, our study demonstrates that solely focusing on land use and climate change while neglecting the adverse impacts of N deposition may lead to an underestimation of biodiversity losses (Roth et al., 2021), including in highly diverse arthropod assemblages that are key for a sustainable agricultural production. It appears that increasing proportions of SNH within agricultural landscapes can contribute to mitigating the negative impacts of N deposition, although trade-offs between biodiversity with associated ecosystem services and agricultural production need to be carefully considered. Therefore, it is important to emphasize that, although we have identified a buffering effect of SNH, the benefits for cropland will generally be limited. For cropland region, in addition to conserving or expanding proportions of SNH, measures to control N emissions or N fertilizer application should therefore complement landscape diversification. Still, in line also with previous studies, our results do emphasize that some areas should be left ‘for nature’(Balmford et al., 2005; Hendershot et al., 2020; Shaffer et al., 2002; Tilman et al., 2017) not only to conserve biodiversity and associated ecosystem services (e.g. biological pest control or pollination in landscapes used for food production), but also

to buffer for the potential negative impacts of high N depositions. This systematic approach has gained heightened significance given the documented temporal escalation in N deposition rates (Galloway et al., 2004), particularly in addressing biodiversity conservation challenges posed to arthropod populations.

It is furthermore worth noting that N deposition may impact animal communities at smaller spatial scales than we consider here (Maskell et al., 2010). Our large-scale analysis was limited by the coarse resolution of available N deposition data. Nevertheless, comparison with finer resolution data for one region (Europe) revealed a generally consistent trend in the effect of N deposition on arthropod species biodiversity (Supplement S3, Table S9-S10, with a Pearson correlation coefficient between fine and coarse N deposition data of 0.85). Regardless of which scales were considered, N deposition was generally associated with biodiversity loss (van der Plas et al., 2024). In addition, it is noteworthy that regions experiencing the highest N deposition, including areas of China and India, are severely under-represented in the PREDICTS database. Our results may hence underestimate the negative deposition impact on biodiversity in regions experiencing the highest N depositions. Sampling of plant and arthropod communities from these areas, and the integration of the respective data into the PREDICTS database, is hence urgently needed to fill existing data gaps.

It was furthermore beyond the scope of our study to investigate specific mechanisms among different taxonomic groups that explain their individual responses to increasing N deposition, both in terms of species richness and abundance patterns. Again, future research is needed, potentially under controlled experimental settings that are representative of current and future predicted N deposition levels, to explore and test potential response mechanisms (Nijssen et al., 2017). Additionally, our models' R^2 values were not high (supplementary Table S3-S4), a trend reflected also by earlier studies (Millard et al., 2021; Outhwaite, McCann, et al., 2022). Although previous studies have demonstrated greater variance explained by N deposition model than our model (Stevens et al., 2004; Stevens et al., 2010), the markedly lower explanatory power observed in our study may be attributable to two methodological distinctions: 1) the predominant focus on plant diversity (as opposed to arthropod diversity) in prior

investigations, and 2) the comparatively restricted geographic scope of earlier research. The impact of N deposition on biodiversity being comparatively less significant than that of land use changes and agricultural intensification (Gallego-Zamorano et al., 2022; Payne et al., 2017; Sage, 2020). While even after accounting for many other variables known to affect biodiversity in our models, N deposition was still a significant explanatory variable in our study. Therefore, while N deposition may not be the primary factor compared to others in agricultural systems or globally prioritize N management is also with importance for global biodiversity conservation.

Conclusion

Our study highlights the necessity to address the detrimental effects of N deposition on biodiversity, as N deposition are still rising globally and, according to our findings, are hence posing a significant and increasing threat to arthropod diversity. Failure to preserve and potentially expand SNH remnants across human- dominated landscapes may therefore result not only in significant arthropod diversity declines due to local N deposition, but will also impact diversity-associated ecosystem services like pollination and biological pest control that underpin a sustainable agricultural production. We therefore propose setting minimum SNH targets within production-dominant landscapes, while simultaneously promoting the ecological intensification of the production systems to safeguard biodiversity while securing sustainable yields even in view of N deposition increases (Bommarco et al., 2013; Kleijn et al., 2011; Tscharntke et al., 2021). The impacts of N deposition we have demonstrated in our investigations also clearly highlight the importance of including reduction strategies for these deposition in agricultural production systems and environmental management approaches around the globe.

Acknowledgements

This work was supported by National Natural Science Foundation of China (no.41871186). S.F. was also supported by the China Scholarship Council (202306350102) to study and work with T.N. at CBER, University College London. T.N. was supported by a grant from the Natural Environment Research Council (NE/R010811/1) and by a University Research Fellowship from the Royal Society. We also thank David Makowski from University Paris-Saclay for his assistance with the statistical analysis in the paper.

Author contributions

Y.L. and S.F. designed the research with inputs from J.C.A., T.N., Z.Y. and C.O.. S.F. performed the research and wrote the first paper draft. Y.L., J.C.A., T.N., C.O., Y.Z., and Z.Y. reviewed and extensively edited the manuscript.

Competing interests

The authors declare no competing interests.

Reference

Ackerman, D., Millet, D. B., & Chen, X. (2019). Global Estimates of Inorganic Nitrogen Deposition Across Four Decades. *Global Biogeochemical Cycles*, *33*(1), 100–107. https://doi.org/10.1029/2018GB005990

Balmford, A., Green, Rhys. E., & Scharlemann, J. P. W. (2005). Sparing land for nature: Exploring the potential impact of changes in agricultural yield on the area needed for crop production. *Global Change Biology*, *11*(10), 1594–1605. https://doi.org/10.1111/j.1365-2486.2005.001035.x

Bates, D., Mächler, M., Bolker, B., & Walker, S. (2015). Fitting Linear Mixed-Effects Models Using lme4. *Journal of Statistical Software*, *67*, 1–48. https://doi.org/10.18637/jss.v067.i01

Bebber, D. P. (2021). The gap between atmospheric nitrogen deposition experiments and reality. *Science of the Total Environment*, *801*, 149774. https://doi.org/10.1016/j.scitotenv.2021.149774

Bobbink, R., Braun, S., Nordin, A., Schutz, K., Strengbom, J., Weijters, M., & Tomassen, H. (2010). Empirical N critical loads for natural and semi-natural ecosystems: 2010 update and review. *Background Document, Workshop Noordwijkerhout Http://Www. Rivm. Nl/Thema/Images/FinalDraftCriticalLoads2010_tcm61–47893. Pdf.*

Bobbink, R., Hicks, K., Galloway, J., Spranger, T., Alkemade, R., Ashmore, M., Bustamante, M., Cinderby, S., Davidson, E., Dentener, F., Emmett, B., Erisman, J.-W., Fenn, M., Gilliam, F., Nordin, A., Pardo, L., & De Vries, W. (2010). Global assessment of nitrogen deposition effects on terrestrial plant diversity: A synthesis. *Ecological Applications*, *20*(1), 30–59. https://doi.org/10.1890/08-1140.1

Bommarco, R., Kleijn, D., & Potts, S. G. (2013). Ecological intensification: Harnessing ecosystem

services for food security. *Trends in Ecology & Evolution*, *28*(4), 230–238. https://doi.org/10.1016/j.tree.2012.10.012

Borer, E. T., & Stevens, C. J. (2022). Nitrogen deposition and climate: An integrated synthesis. *Trends in Ecology & Evolution*, *37*(6), 541–552. https://doi.org/10.1016/j.tree.2022.02.013

Boutin, M., Corcket, E., Alard, D., Villar, L., Jiménez, J.-J., Blaix, C., Lemaire, C., Corriol, G., Lamaze, T., & Pornon, A. (2017). Nitrogen deposition and climate change have increased vascular plant species richness and altered the composition of grazed subalpine grasslands. *Journal of Ecology*, *105*(5), 1199–1209. https://doi.org/10.1111/1365-2745.12743

CBD. (2020). *Global Biodiversity Outlook 5*. Montreal. https://www.cbd.int/gbo/gbo5/publication/gbo-5-en.pdf

CBD, C. (2022). *The Kunming-Montreal Global Biodiversity Framework*. https://www.cbd.int/doc/c/e6d3/cd1d/daf663719a03902a9b116c34/cop-15-l-25-en.pdf

Ceulemans, T., Hulsmans, E., Ende, W. V., & Honnay, O. (2017). Nutrient enrichment is associated with altered nectar and pollen chemical composition in succisa pratensis moench and increased larval mortality of its pollinator bombus terrestris L. *PLoS One*, *12*(4), e0175160. https://doi.org/10.1371/journal.pone.0175160

Chao, A., Chazdon, R. L., Colwell, R. K., & Shen, T.-J. (2005). A new statistical approach for assessing similarity of species composition with incidence and abundance data. *Ecology Letters*, *8*(2), 148–159. https://doi.org/10.1111/j.1461-0248.2004.00707.x

Chakravarthy, A. K., Kammar, V., & Shashank, P. R. (2016). Arthropods: Evolution and ecology. In A. K. Chakravarthy & S. Sridhara (Eds.), Economic and Ecological Significance of Arthropods in Diversified Ecosystems: Sustaining Regulatory Mechanisms (pp. 1–16). Springer. https://doi.org/10.1007/978-981-10-1524-3_1

Cheng, Y., Wang, J., Chang, S. X., Cai, Z., Müller, C., & Zhang, J. (2019). Nitrogen deposition affects both net and gross soil nitrogen transformations in forest ecosystems: A review. *Environmental Pollution*, *244*, 608–616. https://doi.org/10.1016/j.envpol.2018.10.054

Clark, C. M., Cleland, E. E., Collins, S. L., Fargione, J. E., Gough, L., Gross, K. L., Pennings, S. C., Suding, K. N., & Grace, J. B. (2007). Environmental and plant community determinants of species loss following nitrogen enrichment. *Ecology Letters*, *10*(7), 596–607. https://doi.org/10.1111/j.1461-0248.2007.01053.x

Davidson, E. A., David, M. B., Galloway, J. N., Goodale, C. L., Haeuber, R., Harrison, J. A., Howarth, R. W., Jaynes, D. B., Lowrance, R. R., Nolan, B. T., Peel, J. L., Pinder, R. W., Porter, E., & Snyder, C. S. (2012). Excess nitrogen in the U.S. environment: Trends, risks, and solutions. *Issues in Ecology*, *15*. https://rex.libraries.wsu.edu/esploro/outputs/journalArticle/Excess-Nitrogen-in-the-US-Environment/99900667382401842

Deng, O., Zhang, S., Deng, L., Lan, T., Luo, L., Gao, X., & Zhou, W. (2019). Atmospheric dry nitrogen deposition and its relationship with local land use in a high nitrogen deposition region. *Atmospheric Environment*, *203*, 114–120. https://doi.org/10.1016/j.atmosenv.2018.12.037

Dragosits, U., Theobald, M. R., Place, C. J., ApSimon, H. M., & Sutton, M. A. (2006). The potential for spatial planning at the landscape level to mitigate the effects of atmospheric ammonia deposition. *Environmental Science & Policy*, *9*(7–8), 626–638. https://doi.org/10.1016/j.envsci.2006.07.002

Fangmeier, A., Hadwiger-Fangmeier, A., Van Der Eerden, L., & Jäger, H.-J. (1994). Effects of atmospheric ammonia on vegetation—A review. *Environmental Pollution*, *86*(1), 43–82. https://doi.org/10.1016/0269-7491(94)90008-6

Gallego-Zamorano, J., Huijbregts, M. A. J., & Schipper, A. M. (2022). Changes in plant species richness due to land use and nitrogen deposition across the globe. *Diversity and Distributions*, *28*(4), 745–755. https://doi.org/10.1111/ddi.13476

Galloway, J. N., Dentener, F. J., Capone, D. G., Boyer, E. W., Howarth, R. W., Seitzinger, S. P., Asner, G. P., Cleveland, C. C., Green, P. A., Holland, E. A., Karl, D. M., Michaels, A. F., Porter, J. H., Townsend, A. R., & Vöosmarty, C. J. (2004). Nitrogen Cycles: Past, Present, and Future. *Biogeochemistry*, *70*(2), 153–226. https://doi.org/10.1007/s10533-004-0370-0

Galloway, J. N., Townsend, A. R., Erisman, J. W., Bekunda, M., Cai, Z., Freney, J. R., Martinelli, L. A., Seitzinger, S. P., & Sutton, M. A. (2008). Transformation of the Nitrogen Cycle: Recent Trends, Questions, and Potential Solutions. *Science*, *320*(5878), 889–892. https://doi.org/10.1126/science.1136674

Galmarini, S., Koffi, B., Solazzo, E., Keating, T., Hogrefe, C., Schulz, M., Benedictow, A., Griesfeller, J. J., Janssens-Maenhout, G., Carmichael, G., Fu, J., & Dentener, F. (2017). Technical note: Coordination and harmonization of the multi-scale, multi-model activities HTAP2, AQMEII3, and MICS-Asia3: simulations, emission inventories, boundary conditions, and model output formats. *Atmospheric Chemistry and Physics*, *17*(2), 1543–1555. https://doi.org/10.5194/acp-17-1543-2017

Ganuza, C., Redlich, S., Uhler, J., Tobisch, C., Rojas-Botero, S., Peters, M. K., Zhang, J., Benjamin, C. S., Englmeier, J., Ewald, J., Fricke, U., Haensel, M., Kollmann, J., Riebl, R., Uphus, L., Müller, J., & Steffan-Dewenter, I. (2022). Interactive effects of climate and land use on pollinator diversity differ among taxa and scales. *Science Advances*, *8*(18), eabm9359. https://doi.org/10.1126/sciadv.abm9359

Garibaldi, L. A., Oddi, F. J., Miguez, F. E., Bartomeus, I., & Zhu, C. D. (2020). Working landscapes need at least 20% native habitat. *Conservation Letters*. https://doi.org/10.1111/conl.12773

Guo, J. H., Liu, X. J., Zhang, Y., Shen, J. L., Han, W. X., Zhang, W. F., Christie, P., Goulding, K. W. T., Vitousek, P. M., & Zhang, F. S. (2010). Significant acidification in major Chinese croplands. *Science*, *327*(5968), 1008–1010. https://doi.org/10.1126/science.1182570

Habel, J. C., Segerer, A., Ulrich, W., Torchyk, O., Weisser, W. W., & Schmitt, T. (2016). Butterfly community shifts over two centuries. *Conservation Biology*, *30*(4), 754–762. https://doi.org/10.1111/cobi.12656

Habel, J. C., Trusch, R., Schmitt, T., Ochse, M., & Ulrich, W. (2019). Long-term large-scale decline in relative abundances of butterfly and burnet moth species across south-western Germany. *Scientific Reports*, *9*(1), 14921. https://doi.org/10.1038/s41598-019-51424-1

Haddad, N. M., Haarstad, J., & Tilman, D. (2000). The effects of long-term nitrogen loading on grassland insect communities. *Oecologia*, *124*(1), 73–84. https://doi.org/10.1007/s004420050026

Harris, I., Osborn, T. J., Jones, P., & Lister, D. (2020). Version 4 of the CRU TS monthly high-resolution gridded multivariate climate dataset. *Scientific Data*, *7*(1), Article 1. https://doi.org/10.1038/s41597-020-0453-3

Hendershot, J. N., Smith, J. R., Anderson, C. B., Letten, A. D., Frishkoff, L. O., Zook, J. R., Fukami, T., & Daily, G. C. (2020). Intensive farming drives long-term shifts in avian community

composition. *Nature*, *579*(7799), Article 7799. https://doi.org/10.1038/s41586-020-2090-6

Hengl, T., Mendes De Jesus, J., Heuvelink, G. B. M., Ruiperez Gonzalez, M., Kilibarda, M., Blagotić, A., Shangguan, W., Wright, M. N., Geng, X., Bauer-Marschallinger, B., Guevara, M. A., Vargas, R., MacMillan, R. A., Batjes, N. H., Leenaars, J. G. B., Ribeiro, E., Wheeler, I., Mantel, S., & Kempen, B. (2017). SoilGrids250m: Global gridded soil information based on machine learning. *PLOS ONE*, *12*(2), e0169748. https://doi.org/10.1371/journal.pone.0169748

Hicks, W. K., Whitfield, C. P., Bealey, W. J., & Sutton, M. A. (2011). *Nitrogen Deposition and Natura 2000*. https://doi.org/20.500.12592/065g87.

Hijmans, R. J., Van Etten, J., Mattiuzzi, M., Sumner, M., Greenberg, J. A., Lamigueiro, O. P., Bevan, A., Racine, E. B., & Shortridge, A. (2013). Raster package in R. *Version. Https://Mirro Rs. Sjtug. Sjtu. Edu. Cn/Cran/Web/Packa Ges/Rast e r/Raster. Pdf.*

Hoskins, A. J., Bush, A., Gilmore, J., Harwood, T., Hudson, L. N., Ware, C., Williams, K. J., & Ferrier, S. (2016). Downscaling land-use data to provide global 30″ estimates of five land-use classes. *Ecology and Evolution*, *6*(9), 3040–3055. https://doi.org/10.1002/ece3.2104

Hudson, L., Newbold, T., Contu, S., Hill, S. L. L., Lysenko, I., De Palma, A., Phillips, H. R. P., Senior, R. A., Bennett, D. J., Booth, H., Choimes, A., Correia, D. L. P., Day, J., Echeverría-Londoño, S., Garon, M., Harrison, M. L. K., Ingram, D. J., Jung, M., Kemp, V., … Purvis, A. (2014). The PREDICTS database: A global database of how local terrestrial biodiversity responds to human impacts. *Ecology and Evolution*, *4*(24), 4701–4735. https://doi.org/10.1002/ece3.1303

Hudson, L., Newbold, T., Contu, S., Hill, S. L. L., Lysenko, I., Palma, A. D., Phillips, H., Alhusseini, T. I., Bedford, F. E., Bennett, D. J., Booth, H., Burton, V., Chng, W. T. C., Choimes, A., Correia, D. L. P., Day, J., Echeverría-Londoño, S., Emerson, S. R., Gao, D., … Purvis, A. (2016). *The 2016 release of the PREDICTS database* (14). https://doi.org/10.5519/0066354

Hudson, L., Newbold, T., Contu, S., Hill, S. L. L., Lysenko, I., Palma, A. D., Phillips, H., Alhusseini, T. I., Bedford, F. E., Bennett, D. J., Booth, H., Burton, V., Chng, W. T. C., Choimes, A., Correia, D. L. P., Day, J., Echeverría-Londoño, S., Emerson, S. R., Gao, D., … Purvis, A. (2023). *The 2016 release of the PREDICTS database V1.1*. https://doi.org/10.5519/j4sh7e0w

Hudson, L., Newbold, T., Contu, S., Hill, S. L., Lysenko, I., De Palma, A., Phillips, H. R., Alhusseini, T. I., Bedford, F. E., Bennett, D. J., Booth, H., Burton, V. J., Chng, C. W., Choimes, A., Correia, D. L., Day, J., Echeverria-Londono, S., Emerson, S. R., Gao, D., … Purvis, A. (2017). The database of the PREDICTS (Projecting Responses of Ecological Diversity In Changing Terrestrial Systems) project. *Ecol Evol*, *7*(1), 145–188. https://doi.org/10.1002/ece3.2579

Humbert, J.-Y., Dwyer, J. M., Andrey, A., & Arlettaz, R. (2016). Impacts of nitrogen addition on plant biodiversity in mountain grasslands depend on dose, application duration and climate: A systematic review. *Global Change Biology*, *22*(1), 110–120. https://doi.org/10.1111/gcb.12986

Hurtt, G. C., Chini, L. P., Frolking, S., Betts, R. A., Feddema, J., Fischer, G., Fisk, J. P., Hibbard, K., Houghton, R. A., Janetos, A., Jones, C. D., Kindermann, G., Kinoshita, T., Klein Goldewijk, K., Riahi, K., Shevliakova, E., Smith, S., Stehfest, E., Thomson, A., … Wang,

Y. P. (2011). Harmonization of land-use scenarios for the period 1500–2100: 600 years of global gridded annual land-use transitions, wood harvest, and resulting secondary lands. *Climatic Change*, *109*(1), 117. https://doi.org/10.1007/s10584-011-0153-2

Janssens-Maenhout, G., Crippa, M., Guizzardi, D., Dentener, F., Muntean, M., Pouliot, G., Keating, T., Zhang, Q., Kurokawa, J., Wankmüller, R., Denier van der Gon, H., Kuenen, J. J. P., Klimont, Z., Frost, G., Darras, S., Koffi, B., & Li, M. (2015). HTAP_v2.2: A mosaic of regional and global emission grid maps for 2008 and 2010 to study hemispheric transport of air pollution. *Atmospheric Chemistry and Physics*, *15*(19), 11411–11432. https://doi.org/10.5194/acp-15-11411-2015

Jaureguiberry, P., Titeux, N., Wiemers, M., Bowler, D. E., Coscieme, L., Golden, A. S., Guerra, C. A., Jacob, U., Takahashi, Y., Settele, J., Díaz, S., Molnár, Z., & Purvis, A. (2022). The direct drivers of recent global anthropogenic biodiversity loss. *Science Advances*, *8*(45), eabm9982. https://doi.org/10.1126/sciadv.abm9982

Jones, L., Stevens, C., Rowe, E. C., Payne, R., Caporn, S. J. M., Evans, C. D., Field, C., & Dale, S. (2017). Can on-site management mitigate nitrogen deposition impacts in non-wooded habitats? *Biological Conservation*, *212*, 464–475. https://doi.org/10.1016/j.biocon.2016.06.012

Kleijn, D., Kohler, F., Báldi, A., Batáry, P., Concepción, E. d, Clough, Y., Díaz, M., Gabriel, D., Holzschuh, A., Knop, E., Kovács, A., Marshall, E. j. p, Tscharntke, T., & Verhulst, J. (2009). On the relationship between farmland biodiversity and land-use intensity in Europe. *Proceedings of the Royal Society B: Biological Sciences*, *276*(1658), 903–909. https://doi.org/10.1098/rspb.2008.1509

Kleijn, D., Rundlöf, M., Scheper, J., Smith, H. G., & Tscharntke, T. (2011). Does conservation on farmland contribute to halting the biodiversity decline? *Trends in Ecology & Evolution*, *26*(9), 474–481. https://doi.org/10.1016/j.tree.2011.05.009

Klein, A.-M., Vaissière, B. E., Cane, J. H., Steffan-Dewenter, I., Cunningham, S. A., Kremen, C., & Tscharntke, T. (2007). Importance of pollinators in changing landscapes for world crops. *Proceedings of the Royal Society B: Biological Sciences*, *274*(1608), 303–313. https://doi.org/10.1098/rspb.2006.3721

Kormann, U., Rösch, V., Batáry, P., Tscharntke, T., Orci, K. M., Samu, F., & Scherber, C. (2015). Local and landscape management drive trait-mediated biodiversity of nine taxa on small grassland fragments. *Diversity and Distributions*, *21*(10), 1204–1217. https://doi.org/10.1111/ddi.12324

Kuypers, M. M. M., Marchant, H. K., & Kartal, B. (2018). The microbial nitrogen-cycling network. *Nature Reviews Microbiology*, *16*(5), Article 5. https://doi.org/10.1038/nrmicro.2018.9

Le Provost, G., Badenhausser, I., Le Bagousse-Pinguet, Y., Clough, Y., Henckel, L., Violle, C., Bretagnolle, V., Roncoroni, M., Manning, P., & Gross, N. (2020). Land-use history impacts functional diversity across multiple trophic groups. *Proceedings of the National Academy of Sciences*, *117*(3), 1573–1579. https://doi.org/10.1073/pnas.1910023117

Li, Y., Schichtel, B. A., Walker, J. T., Schwede, D. B., Chen, X., Lehmann, C. M. B., Puchalski, M. A., Gay, D. A., & Collett, J. L. (2016). Increasing importance of deposition of reduced nitrogen in the United States. *Proceedings of the National Academy of Sciences*, *113*(21), 5874–5879. https://doi.org/10.1073/pnas.1525736113

Lin, H. P. (2005). Influencing mechanism of the acid rain on the terrestrial plant. *Journal of Fujian*

Forestry Science and Technology, *32*(1), 60.

Lind, E. M., La Pierre, K. J., Seabloom, E. W., Alberti, J., Iribarne, O., Firn, J., Gruner, D. S., Kay, A. D., Pascal, J., Wright, J. P., Yang, L., & Borer, E. T. (2017). Increased grassland arthropod production with mammalian herbivory and eutrophication: A test of mediation pathways. *Ecology*, *98*(12), 3022–3033. https://doi.org/10.1002/ecy.2029

Liu, L., Xu, W., Lu, X., Zhong, B., Guo, Y., Lu, X., Zhao, Y., He, W., Wang, S., Zhang, X., Liu, X., & Vitousek, P. (2022). Exploring global changes in agricultural ammonia emissions and their contribution to nitrogen deposition since 1980. *Proceedings of the National Academy of Sciences*, *119*(14), e2121998119. https://doi.org/10.1073/pnas.2121998119

Liu, X., Duan, L., Mo, J., Du, E., Shen, J., Lu, X., Zhang, Y., Zhou, X., He, C., & Zhang, F. (2011). Nitrogen deposition and its ecological impact in China: An overview. *Environmental Pollution*, *159*(10), 2251–2264. https://doi.org/10.1016/j.envpol.2010.08.002

Lu, X., Jiang, H., Zhang, X., & Jin, J. (2016). Relationship between nitrogen deposition and LUCC and its impact on terrestrial ecosystem carbon budgets in China. *Science China Earth Sciences*, *59*(12), 2285–2294. https://doi.org/10.1007/s11430-015-5277-0

Lv, J., Buerkert, A., Benedict, K. B., Liu, G., Lv, C., & Liu, X. (2019). Comparison of nitrogen deposition across different land use types in agro-pastoral catchments of western China and Mongolia. *Atmospheric Environment*, *199*, 313–322. https://doi.org/10.1016/j.atmosenv.2018.11.021

Maskell, L. C., Smart, S. M., Bullock, J. M., Thompson, K., & Stevens, C. J. (2010). Nitrogen deposition causes widespread loss of species richness in British habitats. *Global Change Biology*, *16*(2), 671–679. https://doi.org/10.1111/j.1365-2486.2009.02022.x

Matson, P., Lohse, K. A., & Hall, S. J. (2002). The Globalization of Nitrogen Deposition: Consequences for Terrestrial Ecosystems. *Ambio*, *31*(2), 113–119. https://doi.org/10.1579/0044-7447-31.2.113

McCLEAN, C. J., van den BERG, L. J. L., Ashmore, M. R., & Preston, C. D. (2011). Atmospheric nitrogen deposition explains patterns of plant species loss. *Global Change Biology*, *17*(9), 2882–2892. https://doi.org/10.1111/j.1365-2486.2011.02462.x

Mehrabi, Z., Ellis, E. C., & Ramankutty, N. (2018). The challenge of feeding the world while conserving half the planet. *Nature Sustainability*, *1*(8), Article 8. https://doi.org/10.1038/s41893-018-0119-8

Mestre, L., Schirmel, J., Hetz, J., Kolb, S., Pfister, S. C., Amato, M., Sutter, L., Jeanneret, P., Albrecht, M., & Entling, M. H. (2018). Both woody and herbaceous semi-natural habitats are essential for spider overwintering in European farmland. *Agriculture, Ecosystems & Environment*, *267*, 141–146. https://doi.org/10.1016/j.agee.2018.08.018

Millard, J., Outhwaite, C. L., Kinnersley, R., Freeman, R., Gregory, R. D., Adedoja, O., Gavini, S., Kioko, E., Kuhlmann, M., Ollerton, J., Ren, Z.-X., & Newbold, T. (2021). Global effects of land-use intensity on local pollinator biodiversity. *Nature Communications*, *12*(1), Article 1. https://doi.org/10.1038/s41467-021-23228-3

Mohamed, A., DeClerck, F., Verburg, P. H., Obura, D., Abrams, J. F., Zafra-Calvo, N., Rocha, J., Estrada-Carmona, N., Fremier, A., Jones, S. K., Meier, I. C., & Stewart-Koster, B. (2024). Securing nature’s contributions to people requires at least 20%–25% (semi-)natural habitat in human-modified landscapes. *One Earth*, *7*(1), 59–71. https://doi.org/10.1016/j.oneear.2023.12.008

Nancy, D., Ashmore, M., Belyazid, S., Bleeker, A., Bobbink, R., W, deVries, Erisman, J. W., Spranger, T., C, S., & Berg, L. (2011). Nitrogen deposition as a threat to European Terrestrial Biodiversity. In *Soil Biology & Biochemistry—SOIL BIOL BIOCHEM* (pp. 463–494).

Neff, F., Korner-Nievergelt, F., Rey, E., Albrecht, M., Bollmann, K., Cahenzli, F., Chittaro, Y., Gossner, M. M., Martínez-Núñez, C., Meier, E. S., Monnerat, C., Moretti, M., Roth, T., Herzog, F., & Knop, E. (2022). Different roles of concurring climate and regional land-use changes in past 40 years' insect trends. *Nature Communications*, *13*(1), Article 1. https://doi.org/10.1038/s41467-022-35223-3

Newbold, T. (2018). predictsFunctions: Functions for reading and processing the PREDICTS data. *R Package Version*, *1*(0), 45–50.

Newbold, T., Hudson, L. N., Hill, S. L. L., Contu, S., Lysenko, I., Senior, R. A., Börger, L., Bennett, D. J., Choimes, A., Collen, B., Day, J., De Palma, A., Díaz, S., Echeverria-Londoño, S., Edgar, M. J., Feldman, A., Garon, M., Harrison, M. L. K., Alhusseini, T., … Purvis, A. (2015). Global effects of land use on local terrestrial biodiversity. *Nature*, *520*(7545), Article 7545. https://doi.org/10.1038/nature14324

Newbold, T., Hudson, L. N., Phillips, H. R. P., Hill, S. L. L., Contu, S., Lysenko, I., Blandon, A., Butchart, S. H. M., Booth, H. L., Day, J., De Palma, A., Harrison, M. L. K., Kirkpatrick, L., Pynegar, E., Robinson, A., Simpson, J., Mace, G. M., Scharlemann, J. P. W., & Purvis, A. (2014). A global model of the response of tropical and sub-tropical forest biodiversity to anthropogenic pressures. *Proceedings of The Royal Society B-Biological Sciences*, *281*(1792), 20141371. https://doi.org/10.1098/rspb.2014.1371

Nijssen, M. E., WallisDeVries, M. F., & Siepel, H. (2017). Pathways for the effects of increased nitrogen deposition on fauna. *Biological Conservation*, *212*, 423–431. https://doi.org/10.1016/j.biocon.2017.02.022

Norris, K. (2008). Agriculture and biodiversity conservation: Opportunity knocks. *Conservation Letters*, *1*(1), 2–11. https://doi.org/10.1111/j.1755-263x.2008.00007.x

Outhwaite, C. L., McCann, P., & Newbold, T. (2022). Agriculture and climate change are reshaping insect biodiversity worldwide. *Nature*, *605*(7908), Article 7908. https://doi.org/10.1038/s41586-022-04644-x

Outhwaite, C. L., Ortiz, A. M. D., Spooner, F. E. B., Dalin, C., & Newbold, T. (2022). Availability and proximity of natural habitat influence cropland biodiversity in forest biomes globally. *Global Ecology and Biogeography*, *31*(8), 1589–1602. https://doi.org/10.1111/geb.13532

Papanikolaou, A. D., Kühn, I., Frenzel, M., & Schweiger, O. (2017). Semi-natural habitats mitigate the effects of temperature rise on wild bees. *Journal of Applied Ecology*, *54*(2), 527–536. https://doi.org/10.1111/1365-2664.12763

Pardo, L. H., Robin-Abbott, M. J., & Driscoll, C. T. (2011). Assessment of N deposition effects and empirical critical loads of nitrogen for ecoregions of the United States. *USDA Forest Service Northern Research Station, Newtown Square, Pennsylvania*, 291.

Payne, R. J., Dise, N. B., Field, C. D., Dore, A. J., Caporn, S. J., & Stevens, C. J. (2017). Nitrogen deposition and plant biodiversity: Past, present, and future. *Frontiers in Ecology and the Environment*, *15*(8), 431–436. https://doi.org/10.1002/fee.1528

Perring, M. P., Bernhardt-Römermann, M., Baeten, L., Midolo, G., Blondeel, H., Depauw, L., Landuyt, D., Maes, S. L., De Lombaerde, E., Carón, M. M., Vellend, M., Brunet, J.,

Chudomelová, M., Decocq, G., Diekmann, M., Dirnböck, T., Dörfler, I., Durak, T., De Frenne, P., … Verheyen, K. (2018). Global environmental change effects on plant community composition trajectories depend upon management legacies. *Global Change Biology*, *24*(4), 1722–1740. https://doi.org/10.1111/gcb.14030

Plassmann, K., Jones, M. L. M., & Edwards-Jones, G. (2010). Effects of long-term grazing management on sand dune vegetation of high conservation interest. *Applied Vegetation Science*, *13*(1), 100–112. https://doi.org/10.1111/j.1654-109X.2009.01052.x

Poggio, S. L., Chaneton, E. J., & Ghersa, C. M. (2010). Landscape complexity differentially affects alpha, beta, and gamma diversities of plants occurring in fencerows and crop fields. *Biological Conservation*, *143*(11), 2477–2486. https://doi.org/10.1016/j.biocon.2010.06.014

Potter, P., Ramankutty, N., Bennett, E. M., & Donner, S. D. (2010). Characterizing the Spatial Patterns of Global Fertilizer Application and Manure Production. *Earth Interactions*, *14*(2), 1–22. https://doi.org/10.1175/2009EI288.1

R Core Team, R. (2013). *R: A language and environment for statistical computing*.

Raven, P. H., & Wagner, D. L. (2021). Agricultural intensification and climate change are rapidly decreasing insect biodiversity. *Proceedings of the National Academy of Sciences*, *118*(2), e2002548117. https://doi.org/10.1073/pnas.2002548117

Rigby, R. A., Stasinopoulos, D. M., & Akantziliotou, C. (2008). A framework for modelling overdispersed count data, including the Poisson-shifted generalized inverse Gaussian distribution. *Computational Statistics & Data Analysis*, *53*(2), 381–393. https://doi.org/10.1016/j.csda.2008.07.043

Roth, T., Kohli, L., Rihm, B., Amrhein, V., & Achermann, B. (2015). Nitrogen deposition and multi-dimensional plant diversity at the landscape scale. *Royal Society Open Science*, *2*(4), 150017. https://doi.org/10.1098/rsos.150017

Roth, T., Kohli, L., Rihm, B., Meier, R., & Amrhein, V. (2021). Negative effects of nitrogen deposition on Swiss butterflies. *Conservation Biology*, *35*(6), 1766–1776. https://doi.org/10.1111/cobi.13744

Rundlöf, M., & Smith, H. G. (2006). The effect of organic farming on butterfly diversity depends on landscape context. *Journal of Applied Ecology*, *43*(6), 1121–1127. https://doi.org/10.1111/j.1365-2664.2006.01233.x

Sage, R. F. (2020). Global change biology: A primer. *Global Change Biology*, *26*(1), 3–30. https://doi.org/10.1111/gcb.14893

Schipper, A. M., Hilbers, J. P., Meijer, J. R., Antão, L. H., Benítez-López, A., Jonge, M. M. J., Leemans, L. H., Scheper, E., Alkemade, R., Doelman, J. C., Mylius, S., Stehfest, E., Vuuren, D. P., Zeist, W., & Huijbregts, M. A. J. (2020). Projecting terrestrial biodiversity intactness with GLOBIO 4. *Global Change Biology*, *26*(2), 760–771. https://doi.org/10.1111/gcb.14848

Schlesinger, W. H. (2009). On the fate of anthropogenic nitrogen. *Proceedings of the National Academy of Sciences*, *106*(1), 203–208. https://doi.org/10.1073/pnas.0810193105

Schwede, D. B., Simpson, D., Tan, J., Fu, J. S., Dentener, F., Du, E., & deVries, W. (2018). Spatial variation of modelled total, dry and wet nitrogen deposition to forests at global scale. *Environmental Pollution*, *243*, 1287–1301. https://doi.org/10.1016/j.envpol.2018.09.084

Semenchuk, P., Plutzar, C., Kastner, T., Matej, S., Bidoglio, G., Erb, K.-H., Essl, F., Haberl, H.,

Wessely, J., Krausmann, F., & Dullinger, S. (2022). Relative effects of land conversion and land-use intensity on terrestrial vertebrate diversity. *Nature Communications*, *13*(1), Article 1. https://doi.org/10.1038/s41467-022-28245-4

Shaffer, M. L., Scott, J. M., & Casey, F. (2002). Noah's Options: Initial Cost Estimates of a National System of Habitat Conservation Areas in the United States. *BioScience*, *52*(5), 439–443. https://doi.org/10.1641/0006-3568(2002)052[0439:NSOICE]2.0.CO;2

Shao, Y., Liu, T., Eisenhauer, N., Zhang, W., Wang, X., Xiong, Y., Liang, C., & Fu, S. (2018). Plants mitigate detrimental nitrogen deposition effects on soil biodiversity. *Soil Biology and Biochemistry*, *127*, 178–186. https://doi.org/10.1016/j.soilbio.2018.09.022

Simkin, S. M., Allen, E. B., Bowman, W. D., Clark, C. M., Belnap, J., Brooks, M. L., Cade, B. S., Collins, S. L., Geiser, L. H., Gilliam, F. S., Jovan, S. E., Pardo, L. H., Schulz, B. K., Stevens, C. J., Suding, K. N., Throop, H. L., & Waller, D. M. (2016). Conditional vulnerability of plant diversity to atmospheric nitrogen deposition across the United States. *Proceedings of the National Academy of Sciences*, *113*(15), 4086–4091. https://doi.org/10.1073/pnas.1515241113

Sirami, C., Gross, N., Baillod, A. B., Bertrand, C., Carrié, R., Hass, A., Henckel, L., Miguet, P., Vuillot, C., Alignier, A., Girard, J., Batáry, P., Clough, Y., Violle, C., Giralt, D., Bota, G., Badenhausser, I., Lefebvre, G., Gauffre, B., … Fahrig, L. (2019). Increasing crop heterogeneity enhances multitrophic diversity across agricultural regions. *Proceedings of the National Academy of Sciences*, *116*(33), 16442–16447. https://doi.org/10.1073/pnas.1906419116

Smart, S. M., Ashmore, M. R., Hornung, M., Scott, W. A., Fowler, D. A., Dragosits, U., Howard, D. C., Sutton, M. A., & Famulari, D. (2004). Detecting the signal of atmospheric N deposition in recent national-scale vegetation change across Britain. *Water, Air, & Soil Pollution: Focus*, *4*, 269–278.

Soroye, P., Newbold, T., & Kerr, J. (2020). Climate change contributes to widespread declines among bumble bees across continents. *Science*, *367*(6478), 685–688. https://doi.org/10.1126/science.aax8591

Stevens, C. J., David, T. I., & Storkey, J. (2018). Atmospheric nitrogen deposition in terrestrial ecosystems: Its impact on plant communities and consequences across trophic levels. *Functional Ecology*, *32*(7), 1757–1769. https://doi.org/10.1111/1365-2435.13063

Stevens, C. J., Duprè, C., Dorland, E., Gaudnik, C., Gowing, D. J. G., Bleeker, A., Diekmann, M., Alard, D., Bobbink, R., Fowler, D., Corcket, E., Mountford, J. O., Vandvik, V., Aarrestad, P. A., Muller, S., & Dise, N. B. (2010). Nitrogen deposition threatens species richness of grasslands across Europe. *Environmental Pollution*, *158*(9), 2940–2945. https://doi.org/10.1016/j.envpol.2010.06.006

Stevens, C. J., Manning, P., van den Berg, L. J. L., de Graaf, M. C. C., Wamelink, G. W. W., Boxman, A. W., Bleeker, A., Vergeer, P., Arroniz-Crespo, M., Limpens, J., Lamers, L. P. M., Bobbink, R., & Dorland, E. (2011). Ecosystem responses to reduced and oxidised nitrogen inputs in European terrestrial habitats. *Environmental Pollution*, *159*(3), 665–676. https://doi.org/10.1016/j.envpol.2010.12.008

Stevens, C. J., Thompson, K., Grime, J. P., Long, C. J., & Gowing, D. J. G. (2010). Contribution of acidification and eutrophication to declines in species richness of calcifuge grasslands along a gradient of atmospheric nitrogen deposition. *Functional Ecology*, *24*(2), 478–484.

https://doi.org/10.1111/j.1365-2435.2009.01663.x

Sutton, M. A., Howard, C. M., Erisman, J. W., Billen, G., Bleeker, A., Grennfelt, P., Van Grinsven, H., & Grizzetti, B. (2011). *The European nitrogen assessment: Sources, effects and policy perspectives*. Cambridge University Press.

Sutton, M. A., Milford, C., Dragosits, U., Place, C. J., Singles, R. J., Smith, R. I., Pitcairn, C. E. R., Fowler, D., Hill, J., ApSimon, H. M., Ross, C., Hill, R., Jarvis, S. C., Pain, B. F., Phillips, V. C., Harrison, R., Moss, D., Webb, J., Espenhahn, S. E., … Wyers, G. P. (1998). Dispersion, deposition and impacts of atmospheric ammonia: Quantifying local budgets and spatial variability. *Environmental Pollution*, *102*(1, Supplement 1), 349–361. https://doi.org/10.1016/S0269-7491(98)80054-7

Tan, J., Fu, J. S., Dentener, F., Sun, J., Emmons, L., Tilmes, S., Sudo, K., Flemming, J., Jonson, J. E., Gravel, S., Bian, H., Davila, Y., Henze, D. K., Lund, M. T., Kucsera, T., Takemura, T., & Keating, T. (2018). Multi-model study of HTAP II on sulfur and nitrogen deposition. *Atmospheric Chemistry and Physics*, *18*(9), 6847–6866. https://doi.org/10.5194/acp-18-6847-2018

Theobald, M. R., Milford, M. C., Hargreaves, M. K. J., Sheppard, M. L. J., Nemitz, M. E., Tang, M. Y. S., Phillips, M. V. R., Sneath, M. R., McCartney, M. L., Harvey, M. F. J., Leith, M. I. D., Cape, M. J. N., Fowler, M. D., & Sutton, M. M. A. (2001). Potential for ammonia recapture by farm woodlands: Design and application of a new experimental facility. *Scientific World Journal*, *1*, 791–801. https://doi.org/10.1100/tsw.2001.338

Throop, H. L., & Lerdau, M. T. (2004). Effects of Nitrogen Deposition on Insect Herbivory: Implications for Community and Ecosystem Processes. *Ecosystems*, *7*(2), 109–133. https://doi.org/10.1007/s10021-003-0225-x

Tilman, D., Clark, M., Williams, D. R., Kimmel, K., Polasky, S., & Packer, C. (2017). Future threats to biodiversity and pathways to their prevention. *Nature*, *546*(7656), Article 7656. https://doi.org/10.1038/nature22900

Tilman, D., Fargione, J., Wolff, B., D'Antonio, C., Dobson, A., Howarth, R., Schindler, D., Schlesinger, W. H., Simberloff, D., & Swackhamer, D. (2001). Forecasting agriculturally driven global environmental change. *Science*, *292*(5515), 281–284. https://doi.org/10.1126/science.1057544

Tscharntke, T., Clough, Y., Wanger, T. C., Jackson, L., Motzke, I., Perfecto, I., Vandermeer, J., & Whitbread, A. (2012). Global food security, biodiversity conservation and the future of agricultural intensification. *Biological Conservation*, *151*(1), 53–59. https://doi.org/10.1016/j.biocon.2012.01.068

Tscharntke, T., Grass, I., Wanger, T. C., Westphal, C., & Batáry, P. (2021). Beyond organic farming – harnessing biodiversity-friendly landscapes. *Trends in Ecology & Evolution*, *36*(10), 919–930. https://doi.org/10.1016/j.tree.2021.06.010

van der Plas, F., Hautier, Y., Ceulemans, T., Alard, D., Bobbink, R., Diekmann, M., Dise, N. B., Dorland, E., Dupré, C., Gowing, D., & Stevens, C. (2024). Atmospheric nitrogen deposition is related to plant biodiversity loss at multiple spatial scales. *Global Change Biology*, *30*(8), e17445. https://doi.org/10.1111/gcb.17445

Vitousek, P. M., Aber, J. D., Howarth, R. W., Likens, G. E., Matson, P. A., Schindler, D. W., Schlesinger, W. H., & Tilman, D. G. (1997). Human Alteration of the Global Nitrogen Cycle: Sources and Consequences. *Ecological Applications*, *7*(3), 737–750.

https://doi.org/10.1890/1051-0761(1997)007[0737:HAOTGN]2.0.CO;2
Vogels, J. J., Verberk, W. C. E. P., Lamers, L. P. M., & Siepel, H. (2017). Can changes in soil biochemistry and plant stoichiometry explain loss of animal diversity of heathlands? *Biological Conservation*, *212*, 432–447. https://doi.org/10.1016/j.biocon.2016.08.039
Wagner, D. L., Fox, R., Salcido, D. M., & Dyer, L. A. (2021). A window to the world of global insect declines: Moth biodiversity trends are complex and heterogeneous. *Proceedings of the National Academy of Sciences*, *118*(2), e2002549117. https://doi.org/10.1073/pnas.2002549117
WallisDeVries, M. F., & Bobbink, R. (2017). Nitrogen deposition impacts on biodiversity in terrestrial ecosystems: Mechanisms and perspectives for restoration. *Biological Conservation*, *212*, 387–389. https://doi.org/10.1016/j.biocon.2017.01.017
Wen, Z., Xu, W., Li, Q., Han, M., Tang, A., Zhang, Y., Luo, X., Shen, J., Wang, W., Li, K., Pan, Y., Zhang, L., Li, W., Collett, J. L., Zhong, B., Wang, X., Goulding, K., Zhang, F., & Liu, X. (2020). Changes of nitrogen deposition in China from 1980 to 2018. *Environment International*, *144*, 106022. https://doi.org/10.1016/j.envint.2020.106022
Zhang, B., Ma, W., Song, L., Liang, X., Xi, X., & Wang, Z. (2023). Nitrogen addition and experimental drought simplified arthropod network in temperate grassland. *Functional Ecology*, *n/a*(n/a). https://doi.org/10.1111/1365-2435.14341
Zuur, A. F., Ieno, E. N., Walker, N., Saveliev, A. A., & Smith, G. M. (2009). *Mixed effects models and extensions in ecology with R*. Springer New York. https://doi.org/10.1007/978-0-387-87458-6

Supplementary

Click here to access/download
Supporting Information
supplementary.docx
