# Supplementary material for: Arthropod biodiversity loss from nitrogen deposition is buffered by natural and semi-natural habitats
Source: PLoS Biol. 2025 Jul 22;23(7):e3003285. doi: 10.1371/journal.pbio.3003285 (PMC12282910; doi:10.1371/journal.pbio.3003285)
Supplement: Attachment — Submitted filename: Response to Reviewers.docx [file pbio.3003285.s025.docx]

# **Dear Editors,**

Thank you for your help with the review process and positive response for our manuscript entitled “Landscape composition mediates global losses in arthropod diversity caused by atmospheric nitrogen deposition” (PBIOLOGY-D-25-00837R1). We would like to also thank the three reviewers for their valuable suggestions and comments, which were of great help in improving the paper.

We have carefully considered and addressed all the reviewers’ comments. The response was marked in blue in the following.

Yours faithfully,

Yunhui Liu (on behalf of all authors)

**--------------------------------------------------------**

# **Reviewer: 1**

In this study, the authors inferred the effects of nitrogen (N) deposition on arthropod diversity by integrating biodiversity data from the PREDICTS database with global N deposition and land cover information. The findings reveal that N deposition negatively impacts arthropod biodiversity, with significant reductions in species richness and abundance, particularly in less anthropogenically modified landscapes.

Thank you for the opportunity to review this interesting study. In my opinion, it is a very well conducted study with clearly described methods and results. The findings are very intresting and support previous studies that demonstrate negative effects of N deposition not only on plants but also on other species groups. I only have few comments:

Thank you for your appreciation of our research and your positive feedback!

**----------------------------------------------------------------**

Main comments

- Based on the presented results, the authors conclude that the negative effect of N deposition can be buffered by a large proportion of semi-natural and natural habitats (SNH) in the surrounding area (see results from line 396). This is an important finding. However, when examining Fig. 3, it appears that high N deposition in areas with a high proportion of SNH even has a positive effect (not just a buffering effect). It is unclear to me whether these are actual effects or could be artefacts. Nevertheless, this is a point that should be discussed in more depth.

Thank you for your comment. Although similar findings have been reported in previous studies, this result requires further validation due to the limited number of sites (only 11 sites) with simultaneously high levels of N deposition and SNH.

To avoid confusion, we have now discussed this point in Line 532-542: “Meanwhile, although SNH in the surrounding landscape can buffer the negative impacts of N deposition, caution is needed not to extrapolate the results beyond the scope of the sampling. It should be noted that high N deposition in areas with a high proportion of SNH even has a positive effect (not just a buffering effect) (Fig 3). The result was in similar to a previous study, which reported the presence of shrubs and N deposition had an interactive effect on soil invertebrate diversity resulting in either an increase or a non-significant increase in invertebrate diversity when shrubs were planted (Shao et al., 2018). However, considering our sampling sites rarely encompassed areas with both high proportions of SHN and high-level N deposition, these positive effects of SNH need to be validated with more field observations in the future.”.

**----------------------------------------------------------------**

- The authors find that the effect of nitrogen deposition is most pronounced in habitats with the least anthropogenic modifications (l. 26-27), and at the same time, an increase in the proportion of natural habitat reduces the negative effect of nitrogen deposition (l. 27-29). To me, this sounded like a contradiction, particularly when first reading the abstract. It should be emphasised more strongly that the former refers to the habitat at the study area, while the latter pertains to the proportion of natural habitat in the surrounding area.

Thank you for pointing this out. we apologize for the confusion caused by the unclear explanation. You are correct — the former refers to the study site, and the latter to the surrounding SNH. We now clarified this point. Line 26-29 now reads: “The strongest negative effects of N deposition on arthropod diversity were observed in locations where the local land use entails the least anthropogenic modification. At the same time, with the exception of cropland-dominated landscapes, increases in the amount of SNH in the surrounding landscape reduced arthropod biodiversity losses associated with N deposition.”.

**----------------------------------------------------------------**

Minor comments

- l. 25: slightly misleading as the N-deposition was not measured in the field at each site.

Thank you for your helpful suggestion. We have changed the word “observed” to “levels” to avoid the misleading in line 24.

**----------------------------------------------------------------**
- l. 28: Add "increases in SNH proportion IN THE SURROUNDINGS reduced ..."

Thank you for your comments, we have added the sentence accordingly.

**----------------------------------------------------------------**
- l.47: Please explain why global-scale effects of N deposition require greater attention.

Thank you for your helpful suggestion. We have re-written this sentence to improve clarity. Line 46-53 now reads: “Although current N deposition has been proposed as a major threat to global terrestrial biodiversity (Jaureguiberry et al., 2022; Payne et al., 2017; Sage, 2020) and has also been identified as one of the five major drivers hindering the achievement of global biodiversity conservation targets (CBD, 2022), its global-scale effects have remained underexplored (Wagner et al., 2021), especially when compared to the increasingly well-recognized impacts land use and climate change have on biodiversity (Ganuza et al., 2022; Neff et al., 2022; Newbold et al., 2015; Outhwaite, McCann, et al., 2022; Semenchuk et al., 2022).”.

**----------------------------------------------------------------**

- l.165-167: The abundance was corrected for the sampling effort. However, such a correction does not appear to have been made for the species richness.

Thank you for your comments. Species richness focuses on the number of species observed within a region, where each species only needs to be detected once to be counted, and its dependency on sampling effort is non-linear and prone to saturation. In contrast, abundance data are more affected due to the variety of methods used to measure abundance across the underlying studies and the variation in measuring it across different taxonomic groups. Abundance metrics are also highly dependent on sampling effort (a relationship that can be assumed to be linear) and require standardization to enable meaningful comparisons. Also See method in Newbold et al. (2015) and Outhwaite et al. (2022).

We added a clarifying sentence, lines 172–174 now reads: “Abundance metrics are highly dependent on sampling effort and require standardization to enable meaningful comparisons. In contrast, the relationship between species richness and sampling effort is non-linear and prone to a saturation effect.”.

References:

Newbold, T., Hudson, L. N., Hill, S. L. L., Contu, S., Lysenko, I., Senior, R. A., Börger, L., Bennett, D. J., Choimes, A., Collen, B., Day, J., De Palma, A., Díaz, S., Echeverria-Londoño, S., Edgar, M. J., Feldman, A., Garon, M., Harrison, M. L. K., Alhusseini, T., … Purvis, A. (2015). Global effects of land use on local terrestrial biodiversity. Nature, 520(7545), Article 7545.

Outhwaite, C. L., McCann, P., & Newbold, T. (2022). Agriculture and climate change are reshaping insect biodiversity worldwide. Nature, 605(7908), Article 7908.

**----------------------------------------------------------------**
- l. 200: Should the N-deposition model be included as a factor in the statistical model?

Thank you for the helpful suggestion. We agree that the choice of N deposition data could influence the results. But high-resolution N deposition data are only available for limited regions rather than globally. Accordingly, we have already built a new model using fine-resolution N deposition datasets in Europe. The trends of results were similar across both datasets (see Supplementary S3), suggesting that the differences between deposition models did not substantially affect our conclusions. Therefore, we did not include the N deposition model as an explicit factor in the statistical model, as doing so would introduce unnecessary complexity without improving model performance or interpretability.

**----------------------------------------------------------------**
- l. 201: Remove Tan et al. in the brackets

Thank you for your comments, we have changed accordingly.

**----------------------------------------------------------------**
- l. 243-244: Check wording of the sentence.

Thank you for point this out, we have re-write this sentence, Line 256-260 now reads: “Notably, N deposition data are closely linked to, and partially derived from, agricultural N fertilizer application (Janssens-Maenhout et al., 2015; Liu et al., 2022). Therefore, the spatial patterns of N deposition and fertilizer application are highly correlated (Kleijn et al., 2009) and can also be used as general intensity indicators of anthropogenic influence on ecosystems.”.

**----------------------------------------------------------------**
- l. 340: The link does not seem to be working.

Thank you for your comments. This may be due to network connectivity issues, which occasionally prevent access to the link. To ensure accessibility, we have downloaded the data and will upload it alongside the code upon the paper's publication.

**----------------------------------------------------------------**
- l. 578: Please add a reference that N deposition is still rising globally.

Thank you for this suggestion, we have added the reference (Galloway et al., 2004; Liu et al., 2022) accordingly (Line 621).
**--------------------------------------------------------**

# **Reviewer: 2**

This article mainly address the how the landscape composition, especially the proportion of natural and semi-natural habitats (SNH), mediate the negative effects of nitrogen deposition on arthropod diversity at a global scale, and the main findings are that N deposition has negative effects on arthropod diversity, and such effect differ across land use types. The most impressive and interesting finding is that the presence and large proportion of SNH in the landscape could offset the negative effects of N deposition on arthropod diversity, which in my view is the novelty of the study. Based on such buffering effect, the authors propose the need to maintain a minimum proportion of natural and semi-natural habitats especially in agricultural landscape, from the perspective of biological conservation and thus the sustainability of ecosystem services. In my view, the strength of the paper lies in its global scale examination and its core finding of buffering effects of SNH for biodiversity loss due to N deposition. However, I still have some major concerns and many minor ones as listed below:

Thank you for your positive feedback and thorough and helpful suggestions!

**----------------------------------------------------------------**
1. The differentiation and the comparison between the effects of reduced N and oxidized N seems a finer question compared to the major theme of the paper. Such differentiation and the comparison may more or less divert readers' attention from your most interesting and novel thematic finding. Especially, in the first scientific question, it come abruptly, as it seems that no backdrop for it was previously set.

Thank you for pointing this out. We agree that the primary objective of our study was not to compare the effects of different forms of N deposition. However, previous research has shown that these forms can indeed have varying impacts on biodiversity. Notably, reduced N tending to exert more negative effects (McClean et al., 2011; Schwede et al., 2018). Reduced N deposition is often associated with agricultural activities, such as N fertilizer input. As population growth continues to drive increased food demand, agriculture may become a major contributor to rising N deposition (Liu et al., 2022). Understanding the role of reduced nitrogen deposition may therefore be particularly important for highlighting the potential negative impacts of N inputs on biodiversity in agricultural landscapes.

To address this point more thoroughly, we have added the following clarification in the Introduction (line 75-80): “Understanding these responses is further complicated by temporal variations in the relative proportions of reduced and oxidized N deposition (Li et al., 2016) can result in significant variations in the ecological effects linked to overall N depositions (Schwede et al., 2018). A better understanding of specific ecological impacts of these two forms of N deposition can support a more targeted and effective biodiversity conservation.”.

Additionally, we have moved the discussion on the effects of the two forms of N deposition to the end of the Discussion section to avoid distracting from the main focus of our study.

Reference:

Liu, L., Xu, W., Lu, X., Zhong, B., Guo, Y., Lu, X., Zhao, Y., He, W., Wang, S., Zhang, X., Liu, X., & Vitousek, P. (2022). Exploring global changes in agricultural ammonia emissions and their contribution to nitrogen deposition since 1980. Proceedings of the National Academy of Sciences, 119(14), e2121998119.

Schwede, D. B., Simpson, D., Tan, J., Fu, J. S., Dentener, F., Du, E., & deVries, W. (2018). Spatial variation of modelled total, dry and wet nitrogen deposition to forests at global scale. Environmental Pollution, 243, 1287–1301.

McCLean, C. J., van den BERG, L. J. L., Ashmore, M. R., & Preston, C. D. (2011). Atmospheric nitrogen deposition explains patterns of plant species loss. Global Change Biology, 17(9), 2882–2892.

**----------------------------------------------------------------**
2. I worry about the match between arthropod assemblage data and land use data, as the former spans 1984-2013, while the latter is from a single year, 2005. After all, the arthropod assemblages would change very quickly following land use conversion, which also happen very abruptly due to human interference.

Thank you for your comments. Data in PREDICTS the database mostly span 1984 to 2013, but the data primarily fall after 2000. The land-use data for the year 2005 were selected because they represent the median sampling year of the biodiversity sampling sites in our study, and the land-use categories are also consistent with those recorded in the PREDICTS database. A similar approach has been used in other PREDICTS-related studies (Ceaușu et al., 2025; Outhwaite et al., 2022).

Reference:

Outhwaite, C. L., McCann, P., & Newbold, T. (2022). Agriculture and climate change are reshaping insect biodiversity worldwide. Nature, 605(7908), Article 7908.

Ceaușu, S., Leclère, D., & Newbold, T. (2025). Geography and availability of natural habitat determine whether cropland intensification or expansion is more detrimental to biodiversity. Nature Ecology & Evolution, 1–16.

**----------------------------------------------------------------**
3. Since you have used data of climate and soil for each site on a global scale, and these data must span a large range, so I am wondering why you did not analyze how the N deposition effect and/or SNH buffering effects were influenced by the climate/soil factors. For example, it is very probably that such effects may differ between dry and wet regions.

Thank you for your comments. We agree that it would be interesting to consider how the N deposition effect and/or SNH buffering effects were influenced by climate/soil factors. In our models, we included interactions between climate and soil factors with N deposition as covariates to improve model accuracy. However, the current model already contains a three-way interaction term—land use × N deposition × SNH. Including additional interactions with climate and soil factors to explore their potential modulation of the buffering effect would result in higher-order interactions, which would make the model overly complex and difficult to interpret. Therefore, considering issues related to model fit and interpretability, we did not include these additional interaction terms.

**----------------------------------------------------------------**
4. The Discussion section is not so well organized, that is, the contents are arranged not in a good logic, and there are quite some contents diverting from the main theme (such as line 556-558). As a consequence, the entire Discussion is not very cohesive. For example, line 432-444, this is about the comparison between the effects of reduced N and oxidized N, which is a finer question compared to the major theme of the paper, but such content was placed in the very beginning of specific discussion. If you maintain this content (please also see my first concern), please postpone it. Moreover, the subsequent part of this paragraph (line 444-459) is about the implication of your results for biodiversity conservation, which should be postponed to the end of Discussion or to a separate section named "Implications", together with other implications such as "conserving or expanding SNH" (line 531-541).

Thank you for our constructive comments. We have restructured the discussion section and removed some content (such as line 556-558) that diverted from the main theme to improve the logical flow. We added four subtitles to make each part clearer. 1.Variation in N deposition impacts across land-use types, 2. Importance of semi-natural and natural habitat, 3. Implication for biodiversity conservation, 4. Limitations and future perspective.

For example, we moved the content comparing the effects of reduced and oxidized nitrogen under the third subtitle. And the section emphasizing the importance of SNH has been moved under the second subtitle.

**----------------------------------------------------------------**
5. You devoted only one paragraph (line 503-521) to discuss the mechanisms of SNH buffering effects, which is in my view far from adequate, because this is your core and novel finding. I suggest to expand this part while briefing other parts especially those that seems too hypothetical or a bit distant from your main theme.

Thank you for your helpful suggestions. We have added a paragraph to the Discussion section addressing the buffering effects of SNH. Line 526-542 now read: “To further enhance the buffering effect, it is important to increase the proportion of SNH in the surrounding landscape. In line also with previous studies, some areas should be left ‘for nature’(Balmford et al., 2005; Hendershot et al., 2020; Shaffer et al., 2002; Tilman et al., 2017) not only to conserve biodiversity and associated ecosystem services (e.g. biological pest control or pollination in landscapes used for food production), but also to buffer for the potential negative impacts of high N depositions. Meanwhile, although SNH in the surrounding landscape can buffer the negative impacts of N deposition, caution is needed not to extrapolate the results beyond the scope of the sampling. It should be noted that high N deposition in areas with a high proportion of SNH even has a positive effect (not just a buffering effect) (Fig 3). The result was in similar to a previous study, which reported the presence of shrubs and N deposition had an interactive effect on soil invertebrate diversity resulting in either an increase or a non-significant increase in invertebrate diversity when shrubs were planted (Shao et al., 2018). However, considering our sampling sites rarely encompassed areas with both high proportions of SHN and high-level N deposition, these positive effects of SNH need to be validated with more field observations in the future.”.

**----------------------------------------------------------------**
Other minor concerns:

1. I suggest the title be changed into "Landscape composition mediates the negative effects of nitrogen deposition on arthropod diveristy"

Thank you for your helpful suggestion. We have changed the title accordingly.

**----------------------------------------------------------------**
2. Line 25, delete "observed"
3. Line 26, for → across
4. Line 42, delete "rates"
5. Line 50, delete ", too"
6. Line 52, of arthropods, the world's most species-rich taxa, are therefore……
7. Line 62, at → for
8. Line 63, exist → coexist
9. Line 67, delete "in response to increased N deposition"
10. Line 68, insert "On the other hand"
11. Line 72, to → for
12. Line 75, delete "that", insert "and" before play
13. Line 80, put forward the comma after "and" to before it
14. Line 94-95, please delete this sentence, as this is another issue beyond this paper
15. Line 104, Consequence → Therefore
16. Line 107, delete "while also"
17. Line 111, insert "and" before challenging
18. Line 120, semi-natural/natural habitats, proportion of cropland → SNH and cropland
19. Line 123, "Differentiated by land-use type" → "Across land-use types"
20. Line 126, extent → proportion
21. Line 130, delete "furthermore"

Thank you for your thorough and helpful suggestions! We have addressed all the aforementioned grammatical and wording issues accordingly.

**----------------------------------------------------------------**
22. Line 130, I don't understand this sentence, "most negative effect" is in comparison with the effects of oxidized N? what do you mean by "not least due to ……"

In general, reduced nitrogen (N) deposition tends to have a more pronounced negative impact on biodiversity due to its role in causing soil acidification. In contrast, oxidized N deposition does not contribute to soil acidification and therefore likely exerts a weaker ecological influence compared to reduced N forms.

In order to make it clearer, we have re-written this sentence (line 134-137): “When comparing different forms of N deposition, reduced N deposition exhibits more significant adverse effects on biodiversity relative to oxidized N deposition, primarily due to its pronounced ability to induce soil acidification.”

**----------------------------------------------------------------**
23. Line 131, the second hypothesis can be changed into "SNH can buffer the negative effect of N deposition on biodiversity"?

Thank you for your helpful suggestion. We have changed the hypothesis accordingly.

**----------------------------------------------------------------**

26. Line 166-167, it is not necessarily the linear relationship, but very probably the asymptote, like the species number vs. area curve.

Thank you for pointing this out. We agree with your point that there is an asymptotic relationship between them. Therefore, to clarify this more effectively, we have added an additional explanation at the end of the sentence. Line 177-181 now read: “We therefore assume that there is a linear relationship between recorded abundance and sampling effort (following Newbold et al., 2014 - it should nonetheless be noted that, for very large samples, this pattern is likely asymptotic once approaching a state where all specimens occurring in a locality have been sampled).”.

**----------------------------------------------------------------**

34. Line 235, MAT and MAP data of only one year 2010? Does it match with the land use data of 2005? And with arthropod data of 1984-2013?

The land-use data for the year 2005 were selected because it represents the median sampling year of the biodiversity sampling sites in our study, and the land-use categories are also consistent with those recorded in the PREDICTS database. A similar approach has been used in other PREDICTS-related studies (Ceaușu et al., 2025; Outhwaite et al., 2022). The year 2010 was chosen for MAT and MAP because the N deposition data correspond to 2010. To maintain consistency with the N deposition data, we also used data from 2010 for MAT and MAP.

We also added a briefly description in Line 270-274 and Line 247-248 to explain it.

Line 270-274 now reads: “We selected the 2005 data as it was closest to the median sampling year of the PREDICTS sites included in our analysis, and the land-use categories are also consistent with those recorded in the PREDICTS database. A similar approach has been used in other PREDICTS-related studies (Ceaușu et al., 2025; Outhwaite et al., 2022).”.

Line 247-258 now reads: “To maintain consistency with the year of N deposition data, the year 2010 was also chosen for MAT and MAP.”.

Reference:

Outhwaite, C. L., McCann, P., & Newbold, T. (2022). Agriculture and climate change are reshaping insect biodiversity worldwide. Nature, 605(7908), Article 7908.

Ceaușu, S., Leclère, D., & Newbold, T. (2025). Geography and availability of natural habitat determine whether cropland intensification or expansion is more detrimental to biodiversity. Nature Ecology & Evolution, 1–16.

**----------------------------------------------------------------**

38. Line 253, which four variables?

Thank you for your comments. In order to make it clearer, we added more description in this sentence. It now reads (line 265-267): “The four variables (MAT, MAP, soil pH and N fertilizer) were extracted based on the geographic coordinates of each sample site.”.

**----------------------------------------------------------------**

24. Line 153, delete "measures of"
25. Line 159, accounted for → encompassed
27. Line 192, from → of
28. Line 202, value should be in singular form
29. Line 207, onto → into
30. Line 213, the semicolon should be comma

31. Line 220-221, can be changed into "we additionally compared the responses of arthropod diversity to the two types of N deposition separately"?
32. Line 226, better combine these two sentences, by insert ", and found that"
33. Line 232, "biodiversity" should be "land use"?
35. Line 241, usage → use
36. Line 243, noted worth → noteworthy
37. Line 249, pH
39. Line 263, based on their relationships
40. Line 270, percentage of
41. Line 276, oxidized and reduced
42. Line 279, to convert these → as to be converted
43. Line 285, with → into
44. Line 300, the interaction effect → its interactive effect

Thank you for your helpful suggestions! We have addressed all the aforementioned grammatical and wording issues accordingly.

**----------------------------------------------------------------**
45. Line 366, Table S3 should be deleted?
46. Line 367, "ranked the second"? in terms of what? Is it possible for both the secondary vegetation and pasture to rank the second? By the way, please insert "of arthropod diversity" after "Negative responses"

47. Line 368, delete "diversity"

Thank you for pointing this out. We have changed this sentence to make it clearer. Line 379-383 now reads: “Species richness also shows significant declines in Secondary vegetation (23%, *p* < 0.001) and Pasture (20%, *p* = 0.011), with both ranking just below Cropland in terms of impact. Negative responses of arthropod species richness to N deposition were observed to be weakest in Plantation forest (only decline 0.7%, *p* < 0.001, Table S3, Fig. 2a).”.

**----------------------------------------------------------------**

48. Line 370-371, the phase "as well as……" is wrongly used, in terms of syntax. How about the primary and secondary vegetation? It is not possible for them to suffer greatest loss as aforementioned pasture!
49. Line 373, compared to that in sites of primary vegetation

Thank you for your helpful suggestions. We have re-written this sentence (line 373-379): “Total abundance also showed varied responses to N deposition among the different land-use types (Fig. 2b, Table S4), with the greatest losses observed in Pasture (decline 65%, *p* < 0.001, Table S4), followed by Primary vegetation and then Secondary vegetation (Fig. 2b, Table S4). The effect of N deposition on total abundance in sites of Cropland (decline 34%, *p* = 0.024, Table S4) and Plantation forest (decline 38%, *p* < 0.001, Table S6) were significantly lower compared to that in sites classed as the other three land-use types.”.

**----------------------------------------------------------------**
51. Line 397-410, in this para, is the first sentence contradictory with the last sentence with respect to the buffering effect of SNH? Moreover, all the words "biodiversity" refer to diversity of arthropods? If so, please specify it as arthropod diversity. There do exist same problems in some other places of the MS.

Sorry for the slight confusion. The buffering effect of SNH was observed in all land-use types except in cropland sites (first sentence). The last sentence referred to the buffering effect of SNH in cropland was not observed, and we have revised it to improve clarity. The last sentence now read (Line 426-428): “Although there was no buffering effect observed for abundance patterns in Cropland (Fig. 3B c), higher percentages of SNH benefited arthropod abundance at least in sites with low levels of N deposition (N deposition < 1000 mg N m^-2^ yr^-1^).”.

In our MS, almost all the words "biodiversity" refers to diversity of arthropods. So, we also changed it to arthropod diversity to make it more precise.

**----------------------------------------------------------------**

52. Line 406-407, this is not a complete sentence!

Thank you for your comments. We have revised this sentence (Line 422-424): “For total abundance, the buffering effects of increased SNH were also observed in sites of pasture, plantation forests, secondary vegetation, and primary vegetation (Fig. 3B a, b, d, e), where a higher percentage of SNH in the surrounding landscape supported a greater number of arthropods.”.

**----------------------------------------------------------------**

50. Line 376, more strong → stronger
53. Line 423, delete "has"
54. Line 426, delete "experiments data"
55. Line 429, furthermore → further
56. Line 436, uniquely liked to → due to
57. Line 438, delete "subsequently"
58. Lien 441, could → might
59. Line 442-43, delete "and" and "has",

Thank you for your helpful suggestions! We have addressed all the aforementioned grammatical and wording issues accordingly.

**----------------------------------------------------------------**
60. Line 447, species richness! But do you mean plant species here, or arthropod species? Please specify!

61. Line 447-448, by grazing that can offset light limitation

Thank you for your comments. These methods are useful for both plant and animal species. We have revised the sentence for clarity (Line 559-563): “Targeted management aimed at enhancing plant and animal diversity could also include biomass removal through harvesting or grazing that mitigates N deposition effects by potentially directly removing some nitrogen, but also by increasing light availability, reducing vegetation height, and promoting heterogeneous microhabitats (Jones et al., 2017; Plassmann et al., 2010; Borer & Stevens, 2022).”.

**----------------------------------------------------------------**
63. Line 457-459, what are such measures?

Thank you for your comments. This measure is referred to “shift in diets”. As it was shown in line 568-570: “Given the high N emissions from animal production, dietary shifts—such as replacing animal- based with plant-based or arthropod-based proteins—may be part of the solution”.

**----------------------------------------------------------------**
62. Line 454, delete "extensive of"

64. Line 465-466, can be changed into: ", their biodiversity may show higher sensitivity to N deposition than plantation forest or pastures"

Thank you for your helpful suggestions. We have changed accordingly.

**----------------------------------------------------------------**
65. Line 468, "reduced edibility of plant resources"? why? Don't plants of higher N content have better edibility？

Thank you for your comments. This error was caused by our imprecise wording. The term "reduced edibility of plant resources" may be misleading; a more accurate description would be a “reduction in plant resources diversity”.

Many animal species feed on specific plant species or species groups, either on fresh biomass, nectar and pollen or seeds, litter and humus (Nijssen et al., 2017). Due to their reliance on single plants species, monophagous animal species are in general more vulnerable to these changes than oligophagous species or non-specialized polyphagous species. Changes in the availability of host plants may occur in different ways: 1) host plants decline so much that densities are insufficient to support a viable population of the dependent herbivore (WallisDeVries, 2004), 2) host plants have changed their phenology and a mismatch in time develops with the phenology of animal species (Visser, 2008), and 3) the physical distance between host plants and other essential resources in the landscape (mating sites, nectar sources other than the host plant) becomes too large (Dennis et al., 2003). These will affect the diversity of plant resources

So, we changed the term "reduced edibility of plant resources" to “reduced plant diversity, which lowers food diversity.” (Line 459-460).

Reference:

Nijssen, M. E., WallisDeVries, M. F., & Siepel, H. (2017). Pathways for the effects of increased nitrogen deposition on fauna. Biological Conservation, 212, 423–431.

WallisDeVries, M. F. (2004). A Quantitative Conservation Approach for the Endangered Butterfly Maculinea alcon. Conservation Biology, 18(2), 489–499. https://doi.org/10.1111/j.1523-1739.2004.00336.x

Visser, M. E. (2008). Keeping up with a warming world; assessing the rate of adaptation to climate change. Proceedings of the Royal Society B: Biological Sciences, 275(1635), 649–659.

Dennis, R. L. H., Shreeve, T. G., & Van Dyck, H. (2003). Towards a functional resource-based concept for habitat: A butterfly biology viewpoint. Oikos, 102(2), 417–426.

**----------------------------------------------------------------**

71. Line 490, plant or arthropod species richness?

Thank you for pointing this out. We have changed the sentence to make it clearer. (line 484-486): “In Central Europe for example, plant species richness in grassland decreased by approximately 23% with increasing N deposition (Stevens et al., 2004), a trend similar to our results for arthropod richness.”.

**----------------------------------------------------------------**

79. Line 514, "as N will not accumulate in the soil", why not?

Thank you for your comments. It is possible that plant-available N was taken up and utilized by the plant community quickly in the plots with planted shrubs, or N addition affected the ecological processes related to N but had no effects on total soil N or soil microbial biomass N. In the absence of planted shrubs, N might have accumulated in the soil due to a lack of nitrogen uptake by plant roots, which in turn may have resulted in a decrease of soil biodiversity (Shao et al., 2018).

We have revised this sentence for clarity (Line 515-518): “Similarly, compared to areas without them, areas with planted shrubs experience less biodiversity loss due to N deposition, as N is taken up and utilized more quickly by the plant community, reducing its direct accumulation in the soil and helping to mitigate biodiversity loss (Shao et al., 2018).”.

Reference:

Shao, Y., Liu, T., Eisenhauer, N., Zhang, W., Wang, X., Xiong, Y., Liang, C., & Fu, S. (2018). Plants mitigate detrimental nitrogen deposition effects on soil biodiversity. Soil Biology and Biochemistry, 127, 178–186.

**----------------------------------------------------------------**

66. Line 470, semicolon → ", for instance,"
67. "per se" should be italicized
68. Line 471, high levels of exposure to N deposition and N application
69. Line 480, delete "direct"
70. Line 483, "but also plantation forests and pasture" → "and this also occurs to plantation forests and pasture"
72. Line 493 could → can
73. Line 498, "providing a reduction in" → "that can shrink"
74. Line 503, delete "the"
75. Line 505, delete "coverage"
76. Line 506, "reduce" → "alleviate the"
77. Line 507, "removing" → "intercepting";
78. Line 513, "from" → "due to"
80. Line 516, "contributes positively towards" → benefit
81. Line 520, "address" → "counteract"
82. Line 521, "N deposition exerts on biodiversity" → "of N deposition on biodiversity"
83. Line 528, "between" → "of"
84. Line 529, "and" → "including"
85. Line 542, delete "furthermore"
86. Line 548, regardless of scales considered
87. Line 556-558, delete this sentence
89. Line 569, being → was
90. Line 571, "accounting for" → "identifying"
91. Line 587, "in view of" → "under the context of"

Thank you for point these out! We have addressed all the aforementioned grammatical and wording issues accordingly.

**----------------------------------------------------------------**

88. Line 559-561, The prospect for future research should be focused on those especially at relevant scale as the present study, or with its combining with manipulative experiments to uncover the underlying mechanisms.

Thank you for your helpful suggestion! We have added one paragraph to highlight the importance of N deposition experiments. Line 607-618: “Future research is needed, under controlled experimental settings that reflect current and projected N deposition levels, to explore and test potential response mechanisms on arthropods, especially considering the cascading effects from changes in plant species (Nijssen et al., 2017). Experimental sampling of arthropod communities is particularly needed in regions with high N deposition, such as China and India. On the other hand, it is also necessary to sample arthropods along gradients of N deposition—a fine-grained observational approach. Compared to high nitrogen additions in controlled experiments, such studies, which are closer to natural environmental conditions-will better support the conservation of species affected by atmospheric N deposition. In particular, comparative experiments between reduced and oxidized N deposition that separate the effects of these two forms will be more beneficial for implementing targeted biodiversity conservation measures.”.

**--------------------------------------------------------**

# **Reviewer: 3**

Reviewer #3:
[identifies himself as David Wagner; IMPORTANT: please see attached file]

This is a much-needed look at the impacts of nitrogen inputs on terrestrial arthropod diversity. Figures are fine. The rigor of the study is reflected in the four supplementary documents. The results make sense, are novel, important, consistent with reports from other studies, and are of global relevance. I suspect that the paper will be highly cited.

I am not sufficiently knowledgeable about the statistical methods to evaluate aspects of the study. Pleased to see that Fan et al. also took mean annual temperature, mean annual precipitation, and soil PH into account (and used variance inflation factors to check for collinearity). Likewise, was pleased that the team analyzed organic oxides separately from reduced nitrogen cmpds.

Most of my comments and suggestions are added to a pdf of the manuscript which I am returning.

Thank you for your positive comments above and the constructive suggestions below. All issues raised in the comments below and in the annotated PDF have been addressed. Please refer to the detailed responses below.

**----------------------------------------------------------------**

**Comments in PDF**

Line 508. This would strike me as minor effect relative to absorption, conversion, and mineralization by a woodland or forest tract.

Mineralization is not mentioned in the manuscript

Thank you for point this out. We added more description about this point in line 565-566: “In European grasslands and woodlands, liming is also commonly used to enhance N mineralization and reduce local N accumulation (Stevens et al., 2013).”.

**----------------------------------------------------------------**

**Comments in PDF**

Line 523. This is a straw man as written. Agency don't focus on just one thing. Conservation biologists understand these matters and that pesticides, invasive species, other forms of pollution, rtc. are in play.

Line 524. Estimation of rates is another matter entirely and does not (so much) depend on one’s beliefs. Rewrite sentence to express that you feel this is a very important stressor that is underappreciated and understudied.

Thank you for your helpful suggestions. To avoid creating a misleading impression, we have revised the sentence to clarify that N deposition needs more attention, rather than implying a lack of awareness or oversimplification.

Line 570-574 now read: “Our study highlights the need to better integrate the impacts of N deposition into biodiversity conservation targets—a factor that warrants greater attention (Wagner et al., 2020), particularly regarding arthropods that are critical to ecosystem functioning and sustainable agriculture (Roth et al., 2021).”.

**----------------------------------------------------------------**

**Other revision in PDF file**

Thank you for your detailed and useful comments. We have changed all the comments accordingly.

**----------------------------------------------------------------**

The Discussion struck me as a bit long and scattershot. Consider shortening and perhaps add a few subsections or header words that organize key points so that readers can find relevant content easily given that the Discussion touches on many subjects.

Thank you for your helpful suggestions. In order to improve the logical flow, we restructured the discussion and added a few sub-headings. 1.Variation in N deposition impacts across land-use types, 2. Importance of semi-natural and natural habitat, 3. Implication for biodiversity conservation, 4. Limitations and future perspective.

For instance, we placed the comparison of reduced vs. oxidized N deposition and their implications for biodiversity under Subtitle 3 (line 549-554). Under Subtitle 2, we focused on the buffering effect of SNH, as well as the necessity of increasing their presence at the regional scale (line 526-542). Also, the part emphasizing the importance of N deposition experiments has been placed under Subtitle 4 (line 607-618).

**----------------------------------------------------------------**
The Introduction and or Discussion need to include text that is broad in scope about how N inputs change the basic ecology of ecosystems: primary productivity, species composition, vegetation structure, litter load, duff characteristics, fire, and perhaps most fundamentally in terrestrial ecosystems, the soil properties (a possible reference https://www.sciencedirect.com/science/article/pii/S0038071722003443), far beyond just pH. As such nitrogen input can and will foundationally trigger changes in the composition of a tract's microfauna, flora, and fauna. And from there tell reader that the Discussion can/will only treat a few examples of such, e.g., some of the effects that have been well studied/documented. This approach would help fix the scattershot feeling I got from reading the Discussion.
Related to the above. Be a bit more even-handed about discussing the consequences of the changes. Not all changes are inherently bad--ecological changes produce losers and winner. For example, it is not only the pests that benefit from N input as implied by the current wording. Likely reckon that at least few imperiled taxa might also do better in a situation with more nitrogen.

Thank you for your comments and great suggestions. We added a brief introduction on how N input affects ecosystems (line 38-42): “N inputs can affect plant primary productivity, species composition, vegetation structure, litter load, duff characteristics, fire regimes, and soil properties (Bobbink, Braun, et al., 2010; Pardo et al., 2011; Wagner, 2020; Frey et al., 2023), which can further reshape biotic communities, impacting microfauna, flora, and fauna globally.”.

We refactored the discussion sections, reordered them, removed some redundancies, and added some new ones to optimize the overall structure. Also, we added some examples to clarity that some winners are positively affected by N deposition (line 497-502):” Especially, N deposition will not only have negative effects on biodiversity, N addition may significantly increase arthropod biomass through compositional turnover, while grassland eutrophication can lead to increases in arthropod abundance (Lind et al., 2017; La Pierre et al., 2016). Moreover, arthropod responses may vary depending on the limiting nutrients in different environments (Nessel et al., 2021).”.

**----------------------------------------------------------------**

The Discussion would benefit from pointing out the there are many terrestrial and freshwater systems globally that are especially N-limited, where atmospheric and dry deposition from the use of the burning of fossil fuels represents an extremely threat to the autochthonous biodiversity. The organisms are specially adapted to survive in such situations, and many are easily displaced once the soils are changed by N inputs…even in wildlands, far away from agriculture and anthropogenic point sources.

It is important to emphasize the need for more experimental studies. This could be a separate paragraph; not be handled as just a sentence or two embedded in a larger paragraph.

We have added a paragraph to the Discussion section addressing the need for more experimental studies. Line 607-618 now read: “Future research is needed, under controlled experimental settings that reflect current and projected N deposition levels, to explore and test potential response mechanisms on arthropods, especially considering the cascading effects from changes in plant species (Nijssen et al., 2017). Experimental sampling of arthropod communities is particularly needed in regions with high N deposition, such as China and India. On the other hand, it is also necessary to sample arthropods along gradients of N deposition—a fine-grained observational approach. Compared to high N additions in controlled experiments, such studies, which are closer to natural environmental conditions-will better support the conservation of species affected by atmospheric N deposition. In particular, comparative experiments between reduced and oxidized N deposition that separate the effects of these two forms will be more beneficial for implementing targeted biodiversity conservation measures.”.

**----------------------------------------------------------------**
Small matters:

* The land use types are capitalized in some places but not others. Edit for uniformity. In this manuscript capitalization might be best.

Thank you for your helpful suggestions. We have revised the text to capitalize all land use type names, ensuring consistency with standard terminology.

**----------------------------------------------------------------**
* Text sometimes equates species richness with biodiversity. That's a little problematic and the authors would be well served by just sticking to using richness. If they are to regard them as synonyms, they should be explicit that they plan to do such somewhere.

Thank you for pointing this out. To clarify, we distinguish species richness from biodiversity. When describing results related to species richness, we use the term 'Species richness' instead of 'biodiversity'. For example, in line 374 and line 380, we changed 'biodiversity' to 'Species richness' for clarity.

**----------------------------------------------------------------**
Lines 40-41: atmospheric deposition as the inclusive term is a misnomer. Come up with another term for pollution that includes agricultural inputs. Maybe anthropogenic inputs, anthropogenic augmentation, or any other word set that less problematically includes atmospheric deposition + fertilizer application(s).

Thank you for your comments. We changed the term “atmospheric N deposition” to “Human-induced N input” in Line 45, to improve the accuracy.

Human-induced N input refers to the inputs of reactive nitrogen in the environment resulting from human activities, such as fertilizer application, fossil fuel combustion, and industrial emissions (Wang et al., 2021).

Reference:

Wang, M., Houlton, B. Z., Wang, S., Ren, C., van Grinsven, H. J., Chen, D., ... & Gu, B. (2021). Human-caused increases in reactive nitrogen burial in sediment of global lakes. The Innovation, 2(4).

**----------------------------------------------------------------**
Lines 64 to 68: might consider citing Wagner, D. L. (2020) [Insect declines in the Anthropocene. Ann. Rev. Entomol. 65: 457-480] here/elsewhere as N pollution received its own subsection in the review. (Text reproduced below.) (Being a biosystematist with training in botany and a rare species biologist, nitrogen pollution scares the pudding out me, and makes me appreciate the gravity of this study.)

Nitrification

The release of atmospheric nitrites from the burning of fossil fuels has the potential to change global ecology and ignite extinction cascades (121, 134). Nitrogen is often a limiting nutrient in terrestrial, freshwater, and marine systems. Many of the world's most speciose and specialized plant communities occur in nitrogen-poor soils (158). In a now-classic study, the generous addition of nitrogen to a pasture at the Rothamsted Experimental Station (in the United Kingdom) resulted in a 10-fold loss of plant richness over the course of the ensuing century (77).

The wet or dry deposition of atmospheric nitrogen to previously nitrogen-limited communities (e.g., bogs, heathlands, sand-based communities, pine barrens, oligotrophic grasslands, and many freshwater ecosystems) will change their fundamental ecology by altering edaphic conditions, nutrient cycling, species interactions and composition, and more. Butterfly declines in northern Europe have been linked to nitrogen deposition, particularly that associated with oligotrophic grasslands (56a, 57, 114).

Soil chemistry is a primary determinant of plant distributions—nitrogen deposition from fossil fuel consumption has the potential to trigger changes in plant communities worldwide. As plant abundance and species composition change, so will the associated entomofaunas. Approximately half of all described insects are plant feeders, and approximately 90% of these are thought to be dietarily specialized herbivores that depend upon just a small suite of closely related species (37, 127, 148). The potential consequences of atmospheric nitrogen deposition are grave and worthy of greater attention.

Thank you for the suggested literature. The referenced literature is highly relevant and has now been cited accordingly (e.g. in line 40, line 572).

**----------------------------------------------------------------**
Line 96: Agriculture (agricultural intensification) is identified as one of the top three drivers of insects declines in Wagner (2020), Wagner et al. (2021), and Raven and Wagner (2021). None of these need be cited…just sayin'.

Thank you for the suggested literature. Following Reviewer 2’s comments, we have deleted this sentence to keep the Introduction more focused on our main aims. However, we have cited these studies elsewhere due to their high relevance to our research. For example, in line 46, line 87, line 106, line 575.

**----------------------------------------------------------------**
Lines 219-220: The effects of the two forms of N (input) do differ—not may differ.

Thank you for pointing this out. We have made the changes accordingly. Furthermore, following the Reviewer 2 's suggestion and to improve clarity, we have moved this sentence to the Introduction section. line 75-80: “Understanding these responses is further complicated by temporal variations in the relative proportions of reduced and oxidized N deposition (Li et al., 2016) can result in significant variations in the ecological effects linked to overall N depositions (Schwede et al., 2018). A better understanding of specific ecological impacts of these two forms of N deposition can support a more targeted and effective biodiversity conservation.”.

**----------------------------------------------------------------**
Lines 243-247: In parts of the USA more fertilizer is deployed on lawns than in croplands! And still further inputs come from impervious surfaces in towns and cities. Not sure these would affect the results as most of the study sites would be outside such areas. But it might be a good idea to analyze the sites within vs away from suburbia and cities, separately, at some point. I am not suggesting that this be done at this time, unless authors were interested in doing so.

Thank you for your insightful and constructive suggestion! Your ideas for our follow-up study provides a reference, which will be the direction of our future efforts.

**----------------------------------------------------------------**
Line 429: There are over 10 million species of insects--why is that a small selection of taxa? Also, as noted above, the issue of nitrogen as a driver is addressed in more detail in Wagner (2020). Wagner et al. (2021) might be deleted here--it would be more relevant to line 79.

Thank you for your helpful suggestions. We have removed this reference here and added the corresponding ones at line 86.

**----------------------------------------------------------------**

Lines 438-439: "subsequently potentially disrupting arthropod trophic networks." This is almost a truism. Every scenario, changes foodwebs: any inputs, withdrawals, time, weather, etc. Somehow this implies to me that these systems are in some stable state that is being disrupted. Everything changes foodwebs; they are inherently dynamic, especially in early successional systems. Consider rephrasing to put something besides the obvious into the statement.

Thank you for your useful suggestion. We have rephrased this sentence and added a relevant reference (Line 549-552): “Reduced (ammonium-dominated) N deposition will therefore alter soil microbial communities, plant secondary metabolites, and plant nutrient ratios more severely than oxidized N deposition, leading to plant damage and reduced plant quality, which in turn can affect arthropod food acquisition (Nijssen et al., 2017).”.

**----------------------------------------------------------------**
Lines 444-459: a bit wordy and scattered--consider tightening this text up...

Thank you for your comments. We have combined some sentences tighten the text.

(line 559-578): “Targeted management aimed at enhancing plant and animal diversity could also include biomass removal through harvesting or grazing that mitigates N deposition effects by potentially directly removing some N, but also by increasing light availability, reducing vegetation height, and promoting heterogeneous microhabitats (Jones et al., 2017; Plassmann et al., 2010; Borer & Stevens, 2022). However, such disturbances may also cause direct mortality or temporarily reduce food resources (Humbert et al., 2009). In European grasslands and woodlands, liming is also commonly used to enhance N mineralization and reduce local N accumulation (Stevens et al., 2013). Furthermore, highly N-efficient agricultural practices and crops, and improved livestock management, may provide additional benefits (Liu et al., 2022). Given the high N emissions from animal production, dietary shifts—such as replacing animal- based with plant-based or arthropod-based proteins—may be part of the solution.”

**----------------------------------------------------------------**
Line 467-468: structural changes can increase or decrease insect diversity. The text is too non-specific. Rework text. Tighten up.

Thank you for your comments. We rewrote this sentence to tighten it (Line 457-462): “As highlighted by previous research (Bobbink, Hicks, et al., 2010; Roth et al., 2021; Stevens et al., 2018), the observed decline in animal diversity caused by N deposition is frequently linked to: (1) reduced plant diversity, which lowers food diversity; (2) increased plant biomass and density, which cool the microclimate and make habitat conditions less suitable for survival; and (3) changes in plant chemical composition, leading to reduced food quality.”.

**----------------------------------------------------------------**
Lines 472-472: As written, the sentence is a truism. Of course, soil pH influences biodiversity… tightened up.

Thank you for pointing this out. We have rephrased this sentence (line 465-468): “Responses to N deposition are further differentiated by differences in soil pH that drives specific plant responses to the depositions, which in turn affects animal species (Stevens et al., 2011; Stevens et al., 2018).”.

**----------------------------------------------------------------**
Lines 508-511: Mineralization is not mentioned in the manuscript. This is a exceedingly important way a grassland, woodland, or forest can remove organic nitrogen from the landscape, and needs to be mentioned somewhere.

We added more description about this point in line 565-566: “In European grasslands and woodlands, liming is also commonly used to enhance N mineralization and reduce local N accumulation (Stevens et al., 2013).”.

**----------------------------------------------------------------**
Lines 523-524: Straw man as written. Agencies/NGO's/governments don't focus on just one thing. Conservation biologists understand these matters to a greater degree than implied here, and that insecticides, herbicides, fungicides, invasive species, other forms of pollution, etc. are all in play.

Line 524: Estimation of rates is another matter entirely and does not (so much) depend on one's beliefs. Perhaps rewrite sentence to express that you feel this important stressor is underappreciated and understudied.

Thank you for your great point. We have rephrased this sentence to make the description more appropriate (Line 570-574): “Our study highlights the need to better integrate the impacts of N deposition into biodiversity conservation targets—a factor that warrants greater attention (Wagner et al., 2020), particularly regarding arthropods that are critical to ecosystem functioning and sustainable agriculture (Roth et al., 2021).”.

**----------------------------------------------------------------**
Question: Might there be latitudinal gradients in fertilizer use (greater use in temperate areas) and in species richness. To what extent are these intertangled or not in this study?

Fig 1. Global map of N fertilizer application (Potter et al., 2010)

Fig 2. Latitudinal profile of N fertilizer application data

Thank you for your valuable question. Actually, there are not a latitudinal gradient in fertilizer use. Detailed in the reference of N fertilizer application data used in our study (Potter et al., 2010): The latitudinal distribution of average fertilizer application rates shows a marked difference north and south of the equator (above Fig 1 and Fig 2). Significantly higher application rates can be found in the Northern Hemisphere, with maxima centered on 30° and 35°N for N and P, respectively. These latitudes correspond to the extensive and intensive agricultural activity found in the United States, western Europe, and China. In the Southern Hemisphere, application rates are generally much lower, with the exception of 7°and 40°S, where high fertilizer use is found in Indonesia and New Zealand’s agricultural areas, respectively.

To compare the relative importance of N fertilizer application versus N deposition, we performed a backward stepwise regression including both variables in the model. The results showed that N deposition had a more significant effect (with N fertilizer application being excluded from the final model), and we also found a high correlation between the N fertilizer and N deposition data (p = 0.64).

There is indeed a latitudinal pattern in species richness, with tropical regions likely harboring higher biodiversity. However, due to data limitations, our model does not include climate zone (tropical and non-tropical) as a variable, which may otherwise introduce high-order interactions and complicate model interpretation. Nonetheless, it would be interesting to consider the difference effect between climate zone, and this represents an important direction for our future work.

Reference:

Potter, P., Ramankutty, N., Bennett, E. M., & Donner, S. D. (2010). Characterizing the Spatial Patterns of Global Fertilizer Application and Manure Production. Earth Interactions, 14(2), 1–22. https://doi.org/10.1175/2009EI288.1

**----------------------------------------------------------------**
As noted above, the Discussion struck me as a tad long, places where adjacent sentences could be combined, authors could dial back on indefinite pronouns, and reword sentences that read as truisms. Polish up a bit to deliver crisp, tight takeaways that readers can digest.

Thank you for your constructive comments. We have streamlined and restructured the discussion section to enhance its clarity and flow.

**----------------------------------------------------------------**
The study is a "satellite's view" of nitrogen deposition and its impacts on insect diversity and abundance. The authors make a pitch for the need of experimental work, which is much needed. But I wonder if there shouldn't also be pitch for studies that fit in between these two scales. For example, local studies that examine insect diversity across nitrogen gradients—a finer-grained approach. Might the authors in their Discussion/Conclusion make a plea for such.

Thank you for your great point. We have added one paragraph and emphasize the necessity of conducting nitrogen deposition gradient experiments under real environmental conditions.

Line 607-618: “Future research is needed, under controlled experimental settings that reflect current and projected N deposition levels, to explore and test potential response mechanisms on arthropods, especially considering the cascading effects from changes in plant species (Nijssen et al., 2017). Experimental sampling of arthropod communities is particularly needed in regions with high N deposition, such as China and India. On the other hand, it is also necessary to sample arthropods along gradients of N deposition—a fine-grained observational approach. Compared to high N additions in controlled experiments, such studies, which are closer to natural environmental conditions-will better support the conservation of species affected by atmospheric N deposition. In particular, comparative experiments between reduced and oxidized N deposition that separate the effects of these two forms will be more beneficial for implementing targeted biodiversity conservation measures.”.

**----------------------------------------------------------------**
In sum, I view this an especially worth study of great relevance to the biodiversity crisis and the matter of insect decline, and I am much in favor of it going forward. Nearly, all my suggestions are merely that and need not be embraced.

Thank you! We sincerely appreciate your positive feedback on our manuscript!

**----------------------------------------------------------------**
